# Frequency matters: how changes in hippocampal theta frequency can influence temporal coding, anxiety-reduction, and memory

**DOI:** 10.3389/fnsys.2022.998116

**Published:** 2023-02-03

**Authors:** Miranda Hines, Steven Poulter, Vincent Douchamps, Francesca Pibiri, Anthony McGregor, Colin Lever

**Affiliations:** Department of Psychology, University of Durham, Durham, United Kingdom

**Keywords:** theta frequency, temporal coding, memory, anxiety, theta slope and y-intercept, hippocampus

## Abstract

Hippocampal theta frequency is a somewhat neglected topic relative to theta power, phase, coherence, and cross-frequency coupling. Accordingly, here we review and present new data on variation in hippocampal theta frequency, focusing on functional associations (temporal coding, anxiety reduction, learning, and memory). Taking the rodent hippocampal theta frequency to running-speed relationship as a model, we identify two doubly-dissociable frequency components: (a) the slope component of the theta frequency-to-stimulus-rate relationship (“theta slope”); and (b) its y-intercept frequency (“theta intercept”). We identify three tonic determinants of hippocampal theta frequency. (1) Hotter temperatures increase theta frequency, potentially consistent with time intervals being judged as shorter when hot. Initial evidence suggests this occurs *via* the “theta slope” component. (2) Anxiolytic drugs with widely-different post-synaptic and pre-synaptic primary targets share the effect of reducing the “theta intercept” component, supporting notions of a final common pathway in anxiety reduction involving the hippocampus. (3) Novelty reliably decreases, and familiarity increases, theta frequency, acting upon the “theta slope” component. The reliability of this latter finding, and the special status of novelty for learning, prompts us to propose a Novelty Elicits Slowing of Theta frequency (NEST) hypothesis, involving the following elements: (1) Theta frequency slowing in the hippocampal formation is a generalised response to novelty of different types and modalities; (2) Novelty-elicited theta slowing is a hippocampal-formation-wide adaptive response functioning to accommodate the additional need for learning entailed by novelty; (3) Lengthening the theta cycle enhances associativity; (4) Even part-cycle lengthening may boost associativity; and (5) Artificial theta stimulation aimed at enhancing learning should employ low-end theta frequencies.

## 1. Introduction

### 1.1 Overview

The literature on the relationships between function and neural oscillations, including hippocampal theta, tends to focus on power, coherence, phase, and cross-frequency coupling. Accordingly, in this article, we focus upon a somewhat neglected topic, variation in hippocampal theta frequency. Arguably, only the relationship between locomotion speed and theta frequency is well-established; the influence of other factors such as novelty, temperature, and anxiolytic drugs on theta frequency are much less studied and/or not widely known. In this article which combines a review of theta frequency related topics, and new data on theta frequency reduction by novelty and anxiolytic drugs, we take the view that the study of theta frequency will provide important clues to how the hippocampal formation supports cognition and emotionality.

We introduce literatures on temperature and interval timing in humans, and on temperature and theta frequency in rodents, to suggest that temperature may provide an interesting avenue by which to explore the relationship between oscillations and timing. This requires a somewhat extended introduction as these literatures are not usually brought together. We describe our previous double dissociation of two constructs of theta frequency, taken from locomotion-based theta in the freely moving rodent: a stimulus-rate (“theta slope”) contribution, and a baseline (“theta intercept”) contribution. We review data from our lab on temperature, novelty, and anxiolytic drugs. Our initial evidence is that temperature acts on the theta slope component. If so, this suggests an approach to evaluate interval timing, whereby flatter theta slopes under colder brain temperatures would correlate with longer estimates of time intervals and steeper slopes with shorter estimates. The robust evidence from our lab suggests that novelty reduces the theta slope component, while anxiolytic drugs reduce the theta intercept component. The finding that widely-different anxiolytic drugs reduce theta intercept suggests that the hippocampus is part of a common final pathway in anxiety reduction, and that emotionality may be more linked to the intercept, than the slope component. Our finding that novelty reduces the theta slope component, and the general finding from several groups that theta frequency is lower in novelty, prompts us to set out a new hypothesis regarding Novelty Elicits Slowing of Theta (NEST) in the hippocampus. We hypothesize that theta frequency slowing in the hippocampal formation is a routine response to novelty which has evolved to accommodate the additional need for learning that novelty calls for. We hypothesize that lengthening the theta cycle functions to enhance associativity, extending to the number and/or sequence of items, and to their memorability over the short and long term.

### 1.2 Why hippocampus and timing?

The hippocampus has long been studied for its support of memory (e.g., O’Keefe and Nadel, 1978; Bannerman et al., 2004) and anxiety (Gray, 1982; Gray and McNaughton, 2000; Bannerman et al., 2004). Recently, there has been a revival in the hippocampal role in temporal coding. Several brain regions contribute to interval timing, notably the striatum (Buhusi and Meck, 2005; Meck, 2005), and there are different kinds of time important to the brain and behaviour. We focus here on the hippocampal contribution in the seconds-to-minutes timescale. Such a focus has at least two foundations. First, understanding the sense of time in the hippocampus is surely crucial to understanding hippocampal-based memory. The discovery of “time cells,” whereby hippocampal pyramidal cells code for temporal stages and/or durations (Pastalkova et al., 2008; MacDonald et al., 2011; Kraus et al., 2013; Salz et al., 2016; Umbach et al., 2020), prompts consideration of timing in hippocampus-dependent sequence memory and episodic memory. Second, behaviour in overt timing tasks appears to be partly controlled by the hippocampus (Meck et al., 1984, 1987; Olton et al., 1987; Vidalaki et al., 1999; Buhusi et al., 2004; Sakata, 2006; Yin and Troger, 2011; Tam and Bonardi, 2012; Barnett et al., 2014; Yin and Meck, 2014; Sabariego et al., 2021; Ross and Easton, 2022). In summary, then, the hippocampus may play some role in explicit assessments of time duration, especially at the multiple-seconds timescale.

### 1.3 Quantal time: the theta cycle as a cognitive moment?

One approach to subjective time is to ask—what defines the unit that is “one moment”? Early psychological theories of perception posited that sensory input was not processed continuously, but quantally in sampling cycles, with each cycle equivalent to one “psychological moment” (e.g., Stroud, 1955; Harter, 1967; O’Hanlon et al., 1974). Stimuli arriving in the same sampling moment would be perceived as simultaneous, and stimuli arriving over more than one moment would be perceived as having duration (O’Hanlon et al., 1974). In human visual perception, the duration of the psychological perceptual moment was estimated as ~54–55 ms (Kristofferson, 1966; O’Hanlon et al., 1974).

In the rodent hippocampus, a candidate hypothesis is that a “cognitive moment” lasts one theta cycle, divided into two approximately-equal halves. Intuitively, the first half consists of a data-driven “outside-in” window opened onto the sensory world, a capture of ground truth (“data”). In the second half, the sensory window shuts, enabling “inside-out” cognition focused on retrieval and prediction functions that can be summarised as “hypothesising.” Simply put, the theta cycle demarcates successive periods of “data, hypothesize; data, hypothesize.” In this section, we first discuss the two halves, and then the equivalence of the whole theta cycle with one cognitive moment.

Theoretical work by Hasselmo and colleagues focused on memory operations in hippocampal region CA1 (Hasselmo et al., 2002) suggests that encoding activity and retrieval activity should be temporally separated to prevent proactive interference (reviewed: Hasselmo, 2011; Easton et al., 2012; Lever et al., 2014). In these models (Hasselmo et al., 2002; Kunec et al., 2005; Cutsuridis and Hasselmo, 2012), the phase of ongoing theta oscillations temporally separates encoding and retrieval, and determines the different plasticity regimes that encoding and retrieval require. Encoding in CA1 occurs preferentially at the peak of pyramidal-layer theta, driven by entorhinal inputs, while CA3-driven retrieval, aided by attractor dynamics, preferentially occurs at the theta trough (Hasselmo et al., 2002). This is consistent with data regarding the timing of entorhinal and CA3 inputs to CA1 (e.g., Mizuseki et al., 2009), with theta phase-dependent synaptic plasticity (e.g., Hyman et al., 2003), and the correlation between memory operations and theta phase of pyramidal cell firing (e.g., Manns et al., 2007; Lever et al., 2010; Jezek et al., 2011; Douchamps et al., 2013; Siegle and Wilson, 2014; Kay et al., 2020; Wang et al., 2020; Poulter et al., 2021). We consistently find that the average theta phase of pyramidal cell spiking in a trial changes significantly according to the dominance of encoding or retrieval (Lever et al., 2010; Douchamps et al., 2013; Poulter et al., 2021) This includes cell-specific changes in different environmental regions within the same trial (Poulter et al., 2021). Using the terms of the sketch above, the encoding phase can be considered as the “data” phase, and the retrieval phase as the “hypothesize” phase.

In one particularly insightful study, two hypothetical scenarios (going to the left or right arm in an alternation task) appeared to be envisaged by the rat hippocampus *“one at a time”* only, with that time being defined by a ~125 ms theta cycle (Kay et al., 2020). As the rat approached the choice point, CA3 and CA1 place cells typically represented each scenario on alternative theta cycles (“left arm, right arm, left arm, right arm…”), before settling on one scenario (“right arm, right arm, right arm”), with this predictive activity consistently occurring at 8 Hz, and in the retrieval half (“hypothesize”) of the theta cycle. This intriguing phenomenon is consistent with theta phase precession, which is ubiquitously observed in hippocampal place cells and entorhinal grid cells in linear tracks (O’Keefe and Recce, 1993; Skaggs et al., 1996), and open fields (Huxter et al., 2008; Climer et al., 2013; Jeewajee et al., 2014). In theta phase precession, firing starts at a later phase of theta, and occurs at progressively earlier phases of the theta cycle, during traversal through a cell’s spatial field. Thus, within a theta cycle, place cells firing at earlier phases have their firing field peaks behind/at the subject’s current location (“perceptual/encoding” phase), while place cells firing at later phases have their firing field peaks ahead of the subject (“predictive/retrieval” phase), perhaps similarly to hypothetical futures.

The Kay et al. (2020) alternation of hypothetical scenarios, one scenario per theta cycle, is reminiscent of Brandon et al. (2013), which observed theta cycle skipping for different head directions, and (Jezek et al., 2011), which also implicated the theta cycle in “one at a time” hypothesising. Jezek et al. (2011) studied CA3 place cell representations “remapping” (Muller and Kubie, 1987; Lever et al., 2002; Leutgeb et al., 2004; Wills et al., 2005) between two different, highly familiar, spatial contexts. In the elegant paradigm of (Jezek et al., 2011) the sensory cue changes used to trigger the two different CA3 representations (“maps”) of the spatial contexts were instantiated *instantaneously*. Following the instantaneous change from one spatial context’s cue configuration to that of the second, ensemble activity flip-flopped between all-or-none maps of either the first or second context, before eventually settling upon the second context. Crucially, the flip-flop transitions occurred on a theta-frequency timescale (~121 ms). That is, mixed maps were rare in a single theta cycle, and especially rare in the second half of a theta cycle (defined by CA3 minimal pyramidal firing), as would be expected if attractor-dynamics based retrieval dominated that half cycle.

This brief review suggests a one-scenario-to-one-theta-cycle equivalence, at least in the rodent hippocampus. Two likely scenarios cannot be conjured up simultaneously, but rather in rapid alternation, and the whole cycle includes one bout of sensory input (“data”: outside-in), as well as one bout of retrieval/prediction (“hypothesize”: inside-out). It follows from this logic that if we want to understand the length of cognitive moments and their rate of passing in the hippocampus, we need to understand factors controlling variation in theta frequency.

### 1.4 The slope vs. intercept components of theta frequency

Cognition-oriented and emotion-oriented accounts of the hippocampus have traditionally assumed an important role for hippocampal theta, and for hippocampal theta frequency in particular (O’Keefe and Nadel, 1978; Gray, 1982; Gray and McNaughton, 2000; McNaughton et al., 2007). Generally, there has been relatively little effort to integrate the hippocampal processing relating to cognition to that relating to emotion, despite their common substrate. One approach has been to say the substrates may be anatomically separate, with one pole (dorsal in rodents, posterior in primates) devoted more to cognition and the other (ventral in rodents, anterior in primates) to emotion, notably anxiety (Bannerman et al., 2004; Pentkowski et al., 2006). However, this anatomical approach may only partly address the functional integration issue because theta has been described as a travelling wave throughout the entire length of the hippocampal long axis in rodents and humans (Lubenov and Siapas, 2009; Patel et al., 2012; Zhang and Jacobs, 2015). One reasonable theoretical starting point is that the processing of cognition should not, of necessity, interfere with the processing of emotion, and *vice versa*. Inspired by the oscillatory-interference model of Burgess (2008), our work (Wells et al., 2013; the present study) explores the potential independence of functional mechanisms associated with what we will call the “theta slope” vs. “theta intercept” components of theta frequency, derived from theta during spontaneous locomotion.

**Figure 1:**
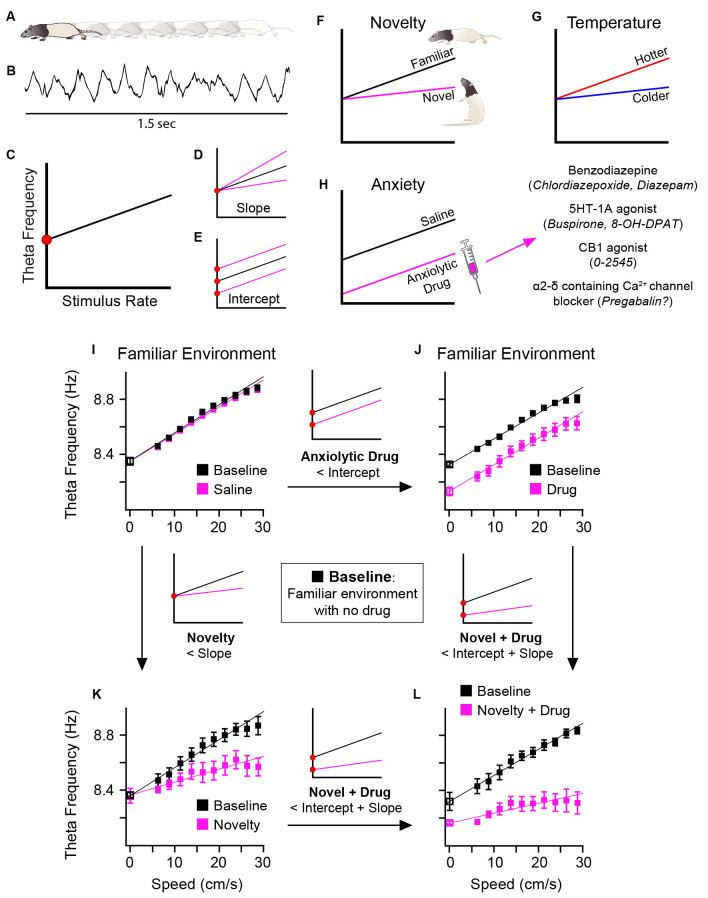
Schematic diagram. Two components of theta frequency and their functional associations. As rats locomote **(A)** during a foraging task, hippocampal formation theta is recorded **(B)**. Taking running speed in such locomotion as a model of stimulus rate, a positive correlation is reliably observed **(C)** between theta frequency (y-axis) and stimulus rate (x-axis). Instantaneous theta frequency is modelled, based on Burgess (2008), as sum of two frequency components: (1) the gain in theta frequency as stimulus rate increases, here called “theta slope” **(D)**; and (2) the “baseline” frequency or y-intercept at zero stimulus rate, here called “theta intercept” **(E)**. Changes in theta slope and theta intercept are doubly dissociable and have different functional associations. Novelty flattens, and familiarity increases, theta slope **(F)**. Temperature positively correlates with theta slope **(G)**. Systemic injection of different classes of anxiolytic drug has a common effect of reducing theta intercept **(H)**. Real data showing double dissociation in effects of novelty and anxiolytic drug upon theta slope and intercept shown in **(I–L)**. In a familiar environment, saline injection has no effect on theta slope for intercept **(I)**, but following injection of an anxiolytic drug, theta intercept is selectively reduced **(J)**. In a novel environment (saline injection as control), theta slope is selectively reduced **(K)**. In a novel environment, following injection of anxiolytic drug, both theta slope and intercept are reduced **(L)**. Parts **(F–H)** schematically summarise data from: dorsal hippocampal theta (Wells et al., 2013; Newman et al., 2013), see Sections “3 Results regarding novelty-familiarity dimension” and “4 The anxiolytic drug pregabalin selectively reduced theta intercept” of the present article; medial entorhinal theta (Monaghan et al., 2017). Parts **(I–L)** adapted from Figure 6 of Wells et al. (2013).

In this article, we present and discuss hippocampal formation theta recorded *from locomoting rats* (Figures 1A,B), taking the speed of locomotion as the model for the stimulus-rate contribution (Figure 1C). The following should be considered as a model of the contributions to the frequency of theta during spontaneous locomotion, not as an empirical characterisation, though we argue for the generalisability of the model. We assume that as the rate of stimuli increases (x-axis), whether sensorily- or intrinsically-driven, so does theta frequency (y-axis; Figure 1C). As regards running speed in rodent, this relationship is broadly linear in our hands (e.g., Wells et al., 2013) in rats over the most common speeds obtained during foraging in an open field (areas up to 1.4 m^2^). Accordingly, this relationship can be modelled using standard linear regression. Encouragingly, this positive frequency-speed correlation has also been found in human hippocampal theta during visually-driven, VR travel (Goyal et al., 2020). Others using visual VR tasks and placing distinct visual cues in the virtual environment, have explicitly compared travel speed vs. cues-per-second and found human hippocampal theta frequency positively correlates with cue rate more than speed (Santos-Pata et al., 2022). In all, broadly speaking, a generalised stimulus-rate contribution to theta frequency seems plausible. Thus, following computational modelling in Burgess (2008), we can now draw attention to two theoretically-different types of change to oscillatory frequency: (1) the stimulus-rate (“theta slope”) contributions to frequency (Figure 1D); and (2) the baseline or y-intercept (“theta intercept”) contributions to frequency (Figure 1E). As discussed further below, these contributions have different correlates or functional associations, theta slope with novelty/familiarity (Figure 1F), and temperature (Figure 1G), and theta intercept with anxiety reduction by systemically-administered anxiolytic drugs (Figure 1H). Burgess (2008) speculated that the “theta slope” component might reflect “Type 1” theta, and the “theta intercept” component “Type 2” theta, but that is not assumed here. In terms of links to behaviour, we note that theta slope (but not theta intercept) negatively predicts the frequency of rearing (Wells et al., 2013), a classic rodent response to novelty (Lever et al., 2006). Importantly, the theta slope and theta intercept components are doubly dissociable (Figures 1I–K), and additive (Figure 1L).

Hippocampal theta, and likely its frequency, appears to support the coding of both space and time (or at least time in a particular context). This “time” coding is not of course a literal approximation of physical clock time, but likely rather of durations and sequences in particular contexts. The oscillatory interference model (O’Keefe and Recce, 1993; Burgess et al., 2007; Burgess, 2008; Hasselmo, 2011) was designed to explain theta phase precession in the spatial domain. In both linear tracks (O’Keefe and Recce, 1993) and open fields (Huxter et al., 2008; Climer et al., 2013; Jeewajee et al., 2014), hippocampal place cells and entorhinal grid cells fire at earlier phases of the theta oscillation as the animal moves through the cell’s spatial field. The basic assumption of oscillatory interference models is that the theta-frequency band rate of one set of inputs impinges on the cell at a slightly faster rate than that of another set of theta-frequency timed inputs. It is the difference between these frequencies that is thought to lead to phase precession. The oscillatory interference model of spatial firing in place and grid cells has been much discussed, and is not our focus here, but we will briefly note studies linking spatial cognition to frequency variation. Jeewajee et al. (2008a) showed the so-called “intrinsic” theta frequency modulation in grid cells increased with running speed and decreased with grid scale. In Wells et al. (2013), we showed a negative correlation between place field size of CA1 place cells and theta slopes in one familiar and two novel environments. Both results are consistent with predictions from the Burgess (2008) model. These correlations were at the whole-trial level. More recently, Dannenberg et al. (2020) showed that grid cell spatial stability correlates with theta slope at smaller temporal scales (10-s segments). Here, perhaps more speculatively, we explore the implications of assuming that running speed’s positive correlation with theta frequency can function as a more general model of stimulus rate’s modulation of theta slope.

Speaking to similarities in coding space and time, it is increasingly clear that time cells recorded in different paradigms also show theta phase precession in the temporal domain, as the animal moves in time through the temporal field (e.g., Pastalkova et al., 2008; Ning et al., 2022). It is important to distinguish between time, distance, and location in time cell experiments (e.g., Kraus et al., 2013; Ning et al., 2022); although this remains to be done in some timing experiments, evidence is consistent with the possibility that when temporal fields stretch for longer duration intervals, the slope of phase precession becomes shallower (Shimbo et al., 2021).

We briefly note an intriguing similarity between the oscillatory interference models originally designed to model phase precession (O’Keefe and Recce, 1993; Burgess et al., 2007; Burgess, 2008), with the striatum-based excitatory-inhibitory beat frequency model of interval timing (Matell and Meck, 2004; Gu et al., 2015). However, our aim here is not to set out, let alone adjudicate between, different oscillatory-related approaches to timing (e.g., Miall, 1989; Matell and Meck, 2004; Hasselmo, 2011; Itskov et al., 2011; Gu et al., 2015; Wang et al., 2015). Rather, our more limited aim is to focus upon arguably-neglected factors controlling *variation* of oscillatory frequency.

**Figure 2:**
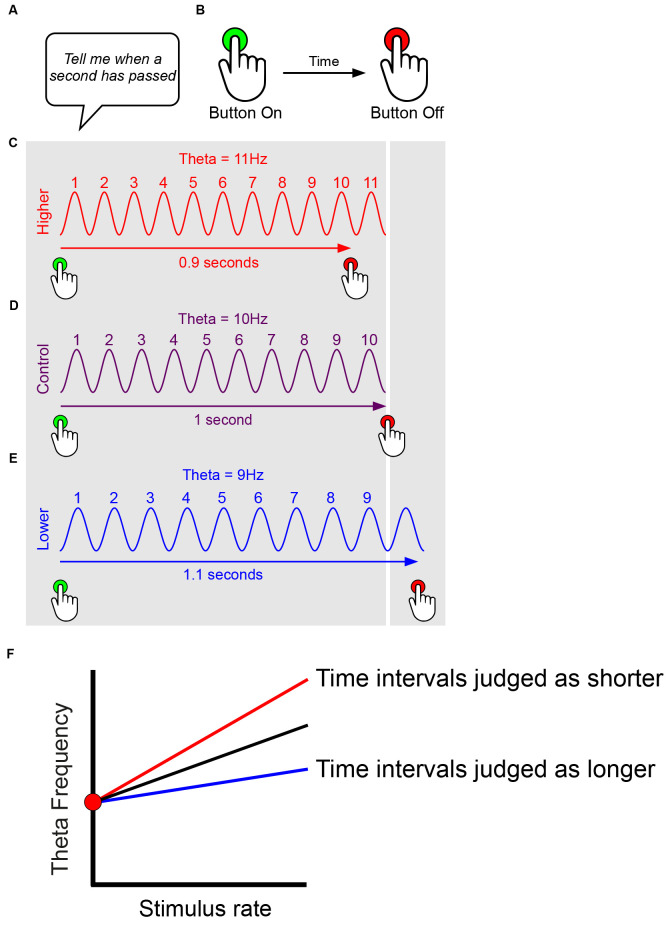
Simplified models suggesting effects of oscillatory frequency upon time interval estimation. This figure presents only simple pace-maker components suitable for interval timing models, and ignores features such as attentional modulation. Experimenter sets up time interval required for rodent/human subject **(A)**. Subject reports time interval, e.g., human presses on and off to match interval required, rodent presses lever etc. **(B)**. Subject’s long-term and recent prior experience (e.g., verbal feedback, reward) sets up basic equivalence of number of oscillatory cycles to time interval, e.g., 10 theta cycles = 1 s. The oscillation can run at a faster pace than usual, such as during a hyperthermic manipulation **(C)**, at a standard pace, such as in a control or baseline condition** (D)**, or at a slower pace than usual, such as during a hypothermic manipulation **(E)**. In the simplest case, when the oscillation runs faster, the subject produces a shorter estimate (e.g., 0.9 s in **C**), because the reference number of cycles (e.g., 10 cycles in **D**) has occurred (accumulated) more quickly; conversely, when the oscillation runs slower, the subject produces a longer estimate (e.g., 1.1 s in **E**), because the reference number of cycles takes longer to accumulate. The above very simple scheme **(C–E)** assumes a constant stimulus rate. However, we presume that oscillatory frequency increases with stimulus rate, and that timing mechanisms likely take account of this, e.g., in order to correct gross biases that would judge time intervals as shorter during running than ambling. Thus, assuming a constant baseline (intercept) frequency, timing may more likely rely on the gradient of the frequency-to-stimulus-rate slope, with time intervals judged as shorter for steeper gradients, and longer for flatter gradients **(F)**.

We first consider temporal coding. In the simplest case of a model relating oscillatory frequency to timing, a single oscillatory signal, functions as the pacemaker (Figure 2). As a result of the subject’s long-term and recent prior experience, which in timing experiments can include verbal feedback and reward, a basic equivalence is set up relating the number of oscillatory cycles to a given time interval, e.g., 10 theta cycles = 1 s. The oscillation can run at a faster pace than usual, such as hippocampal theta during a hyperthermic manipulation (Figure 2C), or at a standard pace, such as in a control or baseline condition (Figure 2D), or at a slower pace than usual, such as during a hypothermic manipulation (Figure 2E). In the simplest case, when the oscillation runs faster, the subject produces a shorter estimate (e.g., 0.9 s in Figure 2C), because the reference number of cycles (e.g., 10 cycles in Figure 2D) has occurred (accumulated) more quickly; conversely, when the oscillation runs slower, the subject produces a longer estimate (e.g., 1.1 s in Figure 2E), because the reference number of cycles takes longer to accumulate. The above very simple scheme assumes a constant stimulus rate.

Importantly, however, we presume that oscillatory frequency increases with stimulus rate, and that timing-related mechanisms may take some account of this over biologically-relevant ranges. For instance, given the well-established modulation of theta frequency by running speed, it is important to correct any gross biases that would judge time intervals as shorter during fast locomotion (running quickly, higher frequencies) than slow locomotion (ambling, lower frequencies). Thus temporal coding may rely not upon the absolute instantaneous frequency of the oscillation, but more likely upon the gradient of the frequency-to-stimulus-rate slope. Under this regime, time intervals would be judged as shorter for steeper gradients, and longer for flatter gradients, as shown in Figure 2F. By this logic, variation in the *y-intercept* of the frequency-to-stimulus-rate relationship would make no difference to timing.

As mentioned above, our focus here is on factors controlling frequency, rather than arguing for one specific oscillatory interference mechanism. A general point is that oscillatory interference approaches that model spatial displacement can be adapted to approaches that model temporal durations (Hasselmo, 2011; Hasselmo and Stern, 2014). What oscillatory interference approaches share is that the greater the difference between the upper and the lower frequency impinging upon the principal cell, the steeper the predicted rate of phase precession whether in place or time and the smaller the predicted spatial field and temporal field. To give one example (Hasselmo and Stern, 2014): a hippocampal time cell’s firing could emerge from two input cells firing at slightly different theta frequencies that begin firing out of phase with each other at the start of a trial. When the input cells fire at the same phase, the time cell fires, and its firing onset codes for the interval of time elapsed since the trial’s start (Figure 6A in Hasselmo and Stern, 2014). If we now assume the higher-frequency input cell has a “theta slope” (stimulus-rate) contribution while its counterpart does not, and theta slope steepens, then the time cell will fire with a quicker latency from the trial onset, with a narrower temporal field. If a population of such time cells supported a time interval judgement, the subject would report time had elapsed more quickly.

#### 1.5 Estimates of time intervals are shorter at higher temperatures: human data

Arguably, the simplest and most reliable finding on the bidirectionality (compression and expansion) of time interval estimation in humans is that time intervals are judged as shorter at higher temperatures and as longer at lower temperatures (Hoagland, 1933; Baddeley, 1966; Hancock, 1993; Aschoff, 1998). This finding is particularly reliable at the seconds to minute timescale.

We first ask—Why are temperature studies important? Much of the literature on time interval estimation is complex and varied in its methods. We would argue that an important feature of the simplest-to-interpret timing studies is the ability to compare the effect of a tonic stimulus, i.e., one that is long-lasting, in a within-subject manner to that obtained from a different level of that tonic stimulus. A comparison of high vs. low temperatures on interval timing provides such an example. In comparison, studies examining responses to briefly-presented stimuli are more complex. As we shall see in a discussion of novelty effects on timing (Section “1.8 Subjective time in novel scenarios—unconsidered effects of theta frequency slowing?” below), it is not necessarily clear to what extent the timing-related response is modulated by the stimulus at the time of its presentation, or by a subsequent after-effect, or by the involvement of remembering the stimulus.

Accordingly, here we first summarise three representative studies looking at the effects of temperature (head, body) upon interval timing in the seconds to minute timescale, where the stimulus (i.e., temperature value) is basically constant throughout the period of the given time estimation trial. Hancock (1993) employed a heating helmet and measured head temperature from the deep auditory meatus (inside the ear). He asked subjects to estimate the passing of 1-s, 11-s, and 41-s durations. When average temperature increased by 0.8°C relative to the placebo condition, estimates were reliably quicker (e.g., 1s: placebo: 1.056 s vs. hot: 0.981 s; 41s: placebo: 46.038 s vs. hot: 40.044 s).

Most temperature-and-timing studies, like Hancock (1993), have used hyperthermic manipulations. Leveraging the readiness of divers to undergo water-induced cooling, Baddeley (1966) found a positive relationship between the rate of counting up to a minute, and oral temperature (median Spearman’s rho: +0.50). On average, when the divers were cooler by 1.3°C, they estimated that a minute had passed about 6 s more slowly (e.g., stopping after 70 s, not 64 s). The study found minimal or absent contributions from heart beat rate, stress, and testing order. Thus, Baddeley found exactly the same relationship as Hancock (1993).

In one particularly rigorous study leveraging natural temperature variation, Aschoff (1998) placed participants in an underground isolation unit for several days, and asked them to estimate 5-s and 10-s intervals. A negative correlation was observed between the rectal temperature and real elapsed time intervals, with an overall mean r across seven subjects of −0.367 (Aschoff, 1998). When a subject was hotter by a few tenths of a centigrade, the subject might reproduce a 10-s interval 1 s faster than under the cooler condition.

In summary, the estimate of a time interval is reliably shorter at higher, and longer at lower, temperatures. This relationship has been widely replicated across various settings such as fever, hot baths, cold diving, exercise, and natural variation (e.g., Hoagland, 1933; Baddeley, 1966; O’Hanlon et al., 1974; Hancock, 1993; Aschoff, 1998; Tamm et al., 2014; van Maanen et al., 2019; Kingma et al., 2021; reviewed Wearden and Penton-Voak, 1995). Some attribute the underlying cause not to higher temperatures *per se* but rather to the higher stress or “arousal” typically elicited by hyperthermic manipulations, e.g., Wearden and Penton-Voak (1995). Importantly, candidate variables such as heart rate were excluded from playing a major role in some of the temperature-related time estimation studies. In Baddeley (1966), while 16/20 divers showed a positive correlation between oral temperature and counting rate, with a median rho of +0.5, only 12/20 divers showed a positive relationship between heart rate and counting rate, and the median rho was only +0.2. In a bath-based study, while increased temperatures produced shorter estimates of durations as usual, increased heart rate did *not* predict *shorter* time estimates, but rather higher *accuracy* of time estimation (Kingma et al., 2021).

In our account, this underestimation of real duration when hotter predicts that the frequency of theta (or other oscillation) should be increased at higher temperatures. If a subjective time mechanism involves, say, counting a reference number of cycles, then these cycles are completed more quickly when the brain is hot.

A more specific prediction arises from oscillatory interference models, and potential similarities between coding of time and space (O’Keefe and Recce, 1993; Burgess et al., 2007; Burgess, 2008; Hasselmo, 2011). The more specific prediction here is that temperature’s positive correlation with theta frequency acts primarily upon *theta slope* (Figures 1C,D). In other words, the specific prediction is that temperature’s increase of theta frequency acts upon what we call the stimulus-rate component of theta frequency (Figure 1G), rather than the baseline (y-intercept) frequency component.

#### 1.6 Higher temperatures increase theta frequency: rodent hippocampal theta

The general form of this frequency-temperature prediction is amply fulfilled. All *in vivo* studies in rodents show a clear, positive relationship between hippocampal theta frequency and brain temperature (or a proxy temperature measure from the head/body—in rodents, body-brain temperature correlations are high; Wells et al., 2013). For instance, this positive relationship between theta frequency and cortical temperature is observed during REM sleep in hamsters, in both euthermic (warm) conditions (combined r values +0.72 and +0.81; Deboer, 2002) and hypothermic conditions (Deboer and Tobler, 1995). In early work, Whishaw and Vanderwolf (1971) observed very strong correlations (averaging *r* = +0.96) between theta frequency and rectal temperature in awake rats, over a very wide range of temperatures (24–42°C).

**Figure 3:**
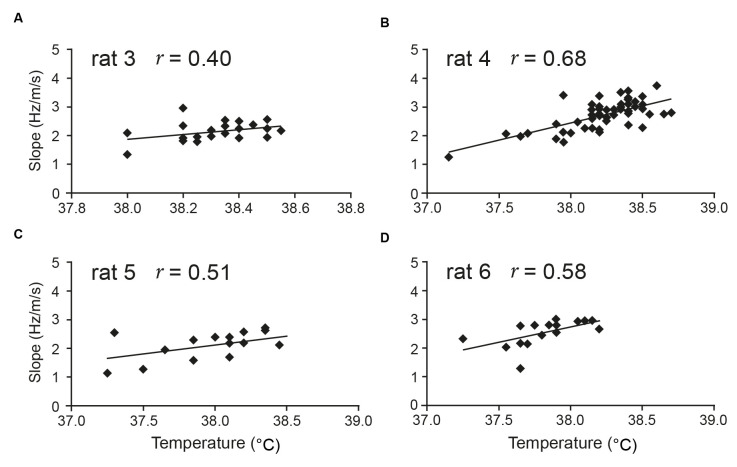
Aural temperature is positively correlated with theta slope. Temperature measured from the ear of rats in a foraging task (average of pre-trial and post-trial) is positively correlated with the slope component (Hz/m/s) of hippocampal theta frequency. In Wells et al. (2013), seven of eight rats tested from two different labs showed a positive correlation between aural temperature and theta slope [four rats’ data shown here: **(A–D)**]. There was no correlation between temperature and theta intercept in any of the eight rats. Figure adapted from Wells et al. (2013).

Our more specific prediction during awake, free behaviour is that temperature positively correlates with theta *slope*. Although the issue clearly requires further study, early indications are indeed that, over a span of around 1–2°C in the 36–39°C range, temperature is particularly positively correlated with theta slope, rather than with theta intercept (Wells et al., 2013). In the latter study, seven out of eight rats locomoting during a foraging task showed a significant positive correlation between aural temperature and theta slope (Figure 3 shows four examples), while none of the eight showed such a relationship for the theta intercept. In summary, while theta-temperature relationships are under-investigated, the general and specific forms of our theta timing account receive support.

Clues regarding possible mechanisms underlying the dissociable theta intercept and theta slope components are provided by observations of carbachol-induced theta in *in vitro* hippocampal slice preparations (Kowalczyk et al., 2001). As mentioned above, all studies from the *in vivo* hippocampus show robust positive correlations between temperature and theta frequency at all temperature ranges measured. In contrast, in the physiological euthermic range of 33–39°C, warming up the artificial CSF reliably increased theta *amplitude*, but not theta *frequency* (Kowalczyk et al., 2001), in the *in vitro* slice. Although other interpretations are possible, this is consistent with the possibility that stimulus-rate mechanisms contributing to theta *slope* variation may be impaired in the *in vitro* slice preparation.

A simple summary of Section “1.5 Estimates of Time intervals are shorter at higher temperatures: human data” is as follows. When rodent brains are hotter, hippocampal theta frequency increases. We suggest a similar relationship obtains in humans, and contributes to human subjects, when hot, making shorter estimates of time intervals.

#### 1.7 Hippocampal theta frequency is lower in mismatch novelty and increases with familiarity

We now turn to a different influence on theta frequency, the novelty/familiarity dimension. Further to our initial finding of theta frequency being lower in environmental novelty (Jeewajee et al., 2008b), we showed in three different settings that this finding arises largely because novelty reduces* theta slope* (Wells et al., 2013, and Figure 1F). Theta slope then increases as the initially novel environment becomes familiar (Wells et al., 2013).

How generalisable is this novelty-frequency reduction effect? We consider the variables of modality (environments vs. objects) and type of novelty (mismatch vs. not-mismatch). Interestingly, the basic result of overall theta frequency reduction by environmental novelty (Jeewajee et al., 2008b) has been replicated in rodents for object novelty (potentially an “object-in-place” novelty). Relative to the theta frequency obtained when exploring a familiar object, theta frequency in control rats (but not Alzheimer’s-Disease-model rats) was *reduced* while exploring a novel object (Villette et al., 2010). Since object exploration often involved rearing on hind legs, it might be objected that perhaps the rearing could reduce theta frequency; however, this is unlikely because rearing typically occurs at rather high theta frequencies (Barth et al., 2018). These initial indications suggest that novelty-elicited frequency reduction may apply to modalities beyond that of spatial contexts.

What about the type of novelty relative to expectations? The novelty explored in the Wells et al. (2013) experiments was a type of *mismatch* contextual novelty, i.e., involving the violation of an expectation of a previously-experienced spatial context. In all of the three novelty-assessing studies in Wells et al. (2013), the novel contexts were located in the same geocentric location as the familiar context; the rats’ hippocampi would expect context A in location X, but instead, find context B in that location. If our account has generality, it should extend beyond mismatch novelty. Accordingly, we studied a non-mismatch version of the novelty-familiarity dimension in the empirical study reported below (Section “3 Results regarding novelty-familiarity dimension”), involving repeated exposures to the same, unchanged context. As we shall see, flatter theta slopes are observed in the first exposures of each day (Section “3.1 The slope component of theta frequency is reduced in non-mismatch type novelty”), suggesting further applicability of the idea that Novelty Elicits Slowing of Theta (NEST). Accordingly, supported by further supporting evidence, we suggest a hypothesis whereby this is not an epiphenomenon, but a hippocampal-wide novelty response enhancing learning (Section “5.7 Implications for plasticity and memory of novelty-elicits slowing of theta (NEST): the NEST hypothesis”).

#### 1.8 Subjective time in novel scenarios—unconsidered effects of theta frequency slowing?

If theta frequency slowing is a general response to novelty, this could affect temporal coding in novelty. Arguably, the empirical base for “psychological” effects upon interval timing by novelty is much less straightforward than that for temperature. One difficulty in anecdotal data is that many novel situations are also more arousing. Another difficulty, which we focus on here, is that experimentally-induced novelty in the lab is much shorter-lasting than changes in temperature, and this complicates interpretation. Our key argument is that if novelty induces slowing of a dominant frequency such as hippocampal theta, with a non-instantaneous onset as seems likely, this could have unforeseen consequences for interval judgements.

To give a flavour of the empirical data, we briefly describe a recent study comparing duration estimates for visual presentations of congruent and incongruent scene-object pairings (Clarke and Porubanova, 2020). Incongruent pairings included objects which were unexpected in the scene context: a woman might be putting not cookies (congruent), but rather a chess board (incongruent), into an oven. Subjects then pressed and released a keyboard bar to reproduce the duration of the stimulus. This study is representative of the generally greater complexity of human novelty-related experiments relative to temperature experiments. In most temperature experiments, subjects are asked to estimate an interval using a verbal, or Start (“press”) and Stop (‘release), method *during* a relatively *long-lasting* manipulation period (“helmet-heated,” “dive-cooled”), basically during a tonic stimulus. In contrast, in novelty experiments, subjects are typically asked to *remember* the duration of a *rather transitory event* that has already passed. This means that their brain activity *in three periods* is potentially relevant to the timing decision, as follows: (1) during the event; (2) during initial recall of that event; and (3) during the time they reproduce the event’s duration.

The subjects’ reproduced durations (Clarke and Porubanova, 2020) were reliably *longer* for the *incongruent scenes*, and this same effect extended to animal stimuli (e.g., incongruent: standard penguin, but with ram’s horns). An additional observation was that this “incongruent = longer” effect occurred whether or not participants were instructed to attend to the incongruence, arguing against an attentional account. We propose that one major factor contributing to the main effect is as follows. The incongruent pairings elicit a novelty-related, slower theta-frequency state in the subjects. This lower-frequency state has a distinct latency but is present by the time the participant reproduces the event’s duration. If the subjects reproduce a “standard” cognitive interval relating to theta frequency, and theta frequency is slower, this would result in a longer duration from press to release of the response button (e.g., 375 ms for three theta cycles running at 8 Hz, compared to 300 ms for three cycles running at 10 Hz).

Overall, the results suggest it is plausible that a state of novelty in humans reduces the frequency of an oscillation such as theta. We suggest that one route to progress in evaluating novelty effects upon timing is to induce a more tonic novelty state, such as being placed in a new, but non-anxiogenic, context for some time.

### 1.9 Anxiolytic drugs reduce the “theta intercept” component (locomotion-based theta)

So far, we have considered two influences on theta frequency, and how they likely act most on theta slope, rather than intercept; higher temperatures and familiarity increasing theta slope. We now look at the other side of the coin; theta intercept, as examined in locomotion-based theta.

Gray and McNaughton (2000) have long noted that all clinically-effective anxiolytic drugs tested to date [i.e., those effective for Generalised Anxiety Disorder (GAD)] reduce the frequency of hippocampal theta elicited by stimulation of the reticular formation. This frequency-reduction effect of reticular-elicited theta is seen across a wide range of anxiolytic drugs, despite their substantial neurochemical dissimilarities (Gray and McNaughton, 2000; McNaughton et al., 2007; Engin et al., 2008; Siok et al., 2009; Yeung et al., 2012, 2013; Young and McNaughton, 2020), but is not seen with *antipsychotic* drugs (Gray and McNaughton, 2000).

We re-examined this frequency-reduction phenomenon *in the theta obtained from spontaneous locomotion* in freely-moving animals. Building upon an oscillatory interference model of grid cells (Burgess, 2008), and the framework in Gray and McNaughton (2000) and McNaughton et al. (2007), we predicted that the theta-frequency reduction effect would act specifically on the “theta intercept” component (Section “1.4 The slope vs. intercept components of theta frequency” above; see Wells et al., 2013; Lever et al., 2014 for discussion). Figures 1E,H,J pictorially summarises previous findings (hippocampal: Wells et al., 2013; entorhinal: Monaghan et al., 2017) whereby different, systemically-administered anxiolytic drugs, reduce the intercept component, but not the slope component, of theta frequency in the freely-moving rat (reviewed Korotkova et al., 2018). This common effect of “theta intercept” reduction is remarkable given the variety of primary neurochemical targets (for an alternative approach to modelling theta frequency based on theta derived from stimulating the reticular formation, see Gray and McNaughton, 2000; Young and McNaughton, 2020).

In this article, we tested our prediction that this “theta intercept” reduction effect would extend to the drug Pregabalin, a drug often used to treat epilepsy and pain, but which is also used, at least in Europe as an anxiolytic drug. Interestingly, Pregabalin has a very different primary target to other anxiolytic drugs, in that it blocks α2-δ-1 subunit-containing voltage-gated calcium channels, and at *presynaptic* sites, providing an interesting challenge to the generality of the “anxiolytic drugs reduce theta intercept” hypothesis. As we shall see (Section “4 The anxiolytic drug pregabalin selectively reduced theta intercept”), this prediction is amply supported.

## 2 Materials and methods

### 2.1 General methods

All experiments were performed under the Animals (Scientific Procedures) Act 1986. Approval for the animal experiments was granted by both Durham University AWERB and the UK Home Office Project and Personal Licenses. When not being tested, all rats were kept in a holding room on a 12–12-h light–dark schedule, with lights off at 2 p.m. Rats were tested during their intended dark phase; however, no attempt was made to prevent any light-driven reset that might have occurred.

All drug injections were given intra-peritoneally for continuity with the previous studies of Wells et al. (2013) and Monaghan et al. (2017), at a volume of 1 ml/kg of body weight. Drugs were dissolved in 0.9% saline. All intraperitoneal injections were performed by a Technician in Durham University’s Life Sciences Support Unit who had very extensive experience with this route of systemic drug administration. All rats were habituated to the experimenter. Each animal was scruffed with a towel daily, following recovery from surgery in the case of the recording experiment, or in the 2–3 days before the anxiolytic drug injection in the case of the anxiety-behaviour test, to get them accustomed to the potentially stressful style of handling for intraperitoneal injections.

### 2.2 Hippocampal theta frequency and place cell recording

#### 2.2.1 Animals

Six male Lister hooded rats (Envigo, Huntington, UK), weighing 300–420 g at the time of surgery, were used as subjects. Food deprivation was maintained during recording periods such that subjects weighed 85–90% of free feeding weight. Water was available *ad libitum*.

#### 2.2.2 Anaesthesia, surgery, screening, and histology

Under deep anesthesia (1–3% isoflurane) and using pre- and post-operative analgesia (buprenorphine, 0.04 mg per kg), rats were chronically implanted with microdrives above the dorsal hippocampus (centroid targets: AP: −3.6 to −4.5; ML: 3.0 mm medio-lateral), with electrodes gradually lowered into the hippocampus over days/weeks. After completion of the experiment, each rat was euthanized with an overdose of sodium pentobarbital and perfused transcardially with saline solution, followed by 4% paraformaldehyde. Brains were sliced coronally into 40-μm-thick sections, which were mounted and stained using cresyl violet solution to aid visualization of electrode tracks and tips.

#### 2.2.3 Electrophysiology and tracking

The microdrives were configured with four tetrodes (16-channel); electrode wires were 25 μm in diameter (90% platinum-10% iridium, California Fine Wire). The furthest distance between tetrodes was generally no more than 400 microns apart. The rats’ head position and orientation were video tracked using two arrays of small LEDs attached to the head-stage. Local field potential (LFP) and neuronal spiking data were recorded using an Axona data acquisition system (Axona, St Albans). LFP signals were recorded in a unipolar fashion with reference to a single ground screw in the temporal skull bone, amplified 4,000–8,000 times, band pass filtered at 0.34–125 Hz, and sampled at 250 Hz.

#### 2.2.4 Apparatus

External cues such as a lamp, PC monitor, and cue cards surrounding the arena provided directional constancy. The testing environment, a wooden square, black-painted, open-field environment (60 × 60 × 50 cm high) with a black Plexiglas floor, was placed on a table elevated 48 cm off the ground. The holding platform, kept away from the testing environment in the corner of the laboratory, was an elevated small wood box (40 × 40 × 30 cm high) which contained woodchip bedding.

#### 2.2.5 Drugs and administration

Pregabalin (35 mg/kg and 17.5 mg/kg; Sigma-Aldrich, Poole, UK) was dissolved in a vehicle of saline solution (0.9% NaCl). Saline only was used in the control condition. The drug solution was prepared on each testing day. The interval between the injection and the following test was 30 min.

The study used two small groups of *n* = 3 at two different doses, which were combined for statistical analysis (*n* = 6): 35 mg/kg, based on behavioural studies (Field et al., 2001; Gambeta et al., 2017), and one which showed reduced theta frequency in reticular-stimulation under-anaesthesia experiment (Siok et al., 2009); 17.5 mg/kg, i.e., exactly half the higher dose, since pregabalin can effectively reduce anxiety behaviour in rodent models at doses as low as 10 mg/kg (Field et al., 2001).

#### 2.2.6 Recording sites

**Figure 4:**
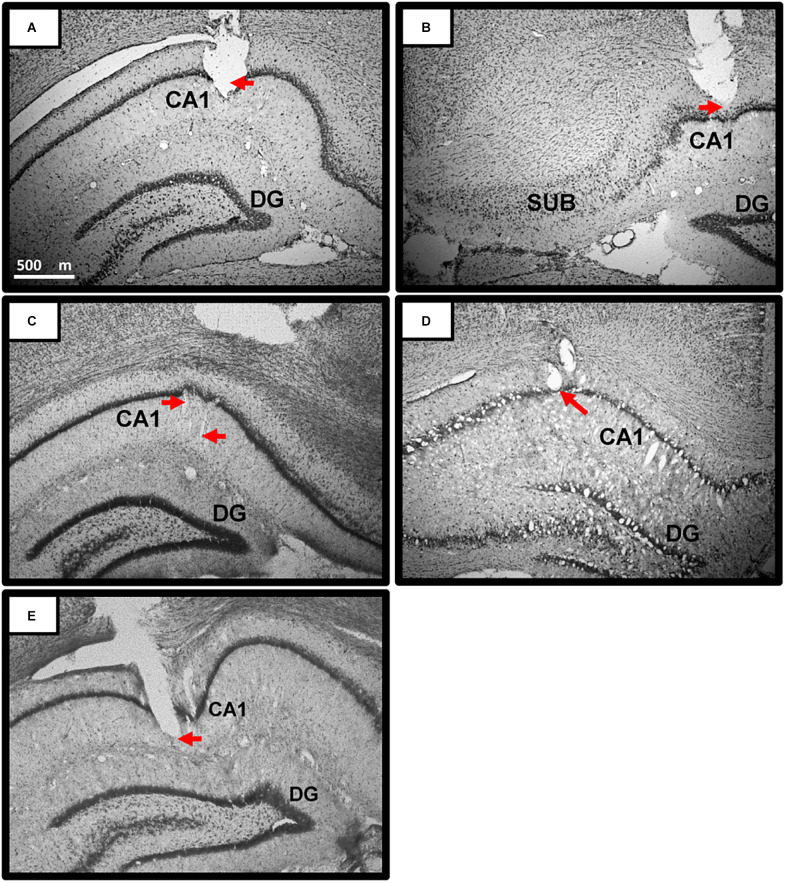
Representative Theta Recording sites in CA1 of dorsal hippocampus. **(A–E)** Photomicrographs of coronal sections depicting representative recording sites in and around the pyramidal layer of the CA 1 region of the dorsal hippocampus. Red arrows point to estimated location of the tetrodes at time of recording. All dorsal-hippocampal tetrodes (four or eight per rat) were used to derive theta intercept and theta slope measures, with intercept and slope measures averaged to give a single value for each rat for each trial. Overall, estimated recording locations included all strata of CA1 (oriens, pyramidale, radiatum, and lacunosum-moleculare) from around −3.0 mm AP to −5.5 mm AP (-AP: i.e., behind bregma) along the anterior-posterior axis of the dorsal hippocampus. Scale bar in **(A)** applies to **(A–E)**. Rats: Rat 3 **(A,B)**; Rat **4 (C)**; Rat 5 **(D)**; Rat 6 **(E)**. Abbreviations: CA1, Cornu Ammonis1; DG, Dentate gyrus; SUB, Subiculum.

**Figure 5:**
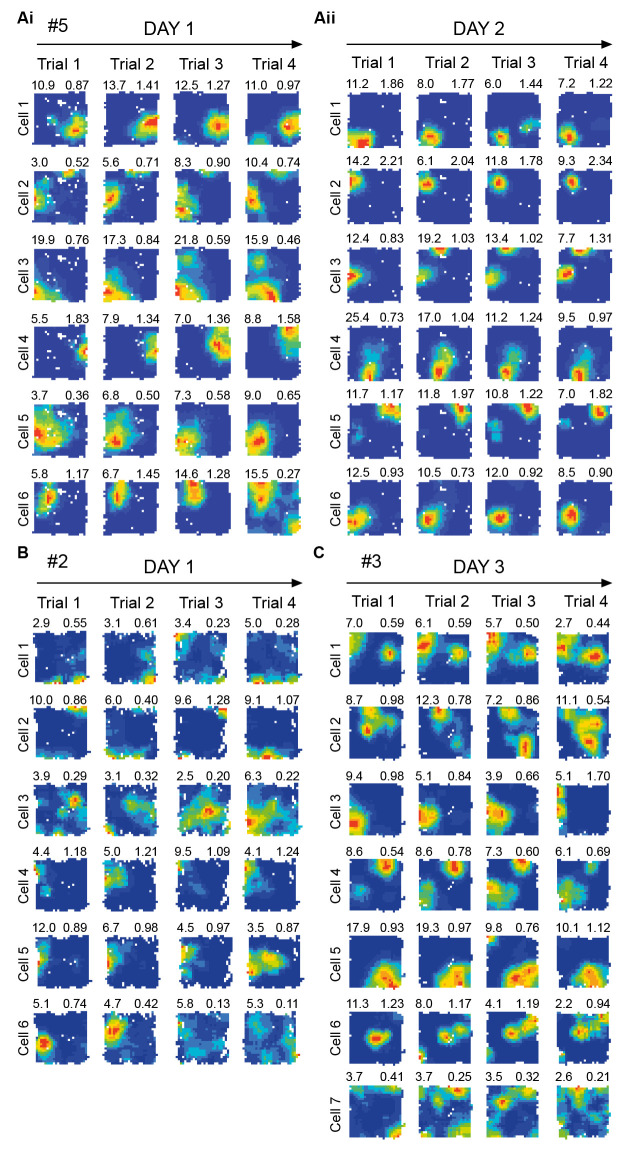
Place cell firing activity during repeated exposure to the same environment. Representative dorsal. CA1 place cells recorded simultaneously from a subset of the electrodes used to record theta frequency variables shown in Figure 6. No obvious novelty/familiarity related pattern, such as in firing rates, is observable in place cells (statistics: see Section “3.2 No significant effects of novelty in dorsal CA1 place cells” text). This is in contrast to the clear novelty/ familiarity related pattern (lower/higher frequency) seen in theta in Figure 6. Each row shows one cell’s locational firing rate maps across trials 1–4 on a given day, with all cells shown for given rat/day combination. Arbitrary cell number shown far left of each row. Locational peak rate (Hz) shown top left, and Skaggs spatial information (bits/spike) shown top right, of each rate map. Cells recorded on Day 1 **(Ai)** and Day 2 **(Aii)** for Rat 5. **(B)** Cells recorded on Day 1 for Rat 2. **(C)** Cells recorded on Day 3 for Rat 3. # denotes rat number.

Our previous work has shown the reported novelty-related theta slope effects at various sites throughout the hippocampal formation (Wells et al., 2013), including CA1, dentate gyrus, and subiculum. Anxiolytic-drug theta intercept effects were tested and shown in different layers of CA1 (Wells et al., 2013). Importantly, our theory is in, and all data to date supports, *global* effects of novelty and anxiolytic drugs on locomotion-derived theta obtained *throughout the hippocampal formation* (e.g., Wells et al., 2013; Monaghan et al., 2017). We did not hypothesise sub-region specific or layer-specific effects, and did not attempt to target specific CA1 layers. Overall, estimated recording locations included all strata of CA1 (oriens, pyramidale, radiatum, and lacunosum-moleculare) from around −3.0 mm AP to −5.5 mm AP (i.e., behind bregma) along the anterior-posterior axis of the dorsal hippocampus (see Figure 4 for examples). No attempt was made to assign precise recording locations for each individual tetrode, or to select a single tetrode for theta analysis. Rather, all dorsal-hippocampal tetrodes (four or eight per rat, six rats) were used to derive theta intercept and theta slope measures, with intercept and slope measures averaged (*n* = 4 or 8) to give a single value for each rat for each trial. Records of tetrode movement and CA1 place cell recording in five of the six rats added to histological identification. For rat 6, place cells were not recorded but histology confirmed sub pyramidal-layer recording sites (e.g., Figure 4). For rats 1 and 2, brains were accidentally disposed of, but place cells were recorded from these rats (e.g., Figure 5) at locations obviously consistent with CA1, and other tetrode tips were ≤400 microns away from those recording place cells.

#### 2.2.7 Place cell recording and analysis

Signals on the channels dedicated to single-cell recording were amplified (10,000–20,000×) and band-pass filtered (500 Hz–7 kHz). All the channels of a given tetrode were recorded differentially with respect to a channel on another tetrode within the same hemisphere. Each channel was monitored at a sampling rate of 50 kHz. Action potentials were stored as 50 points per channel (1 ms, with 200 μs pre-threshold and 800 μs post-threshold) whenever the signal from any of the pre-specified recording channels exceed a given threshold. Recording parameters could be altered across days, but were kept stable within a day. For analysis of place cells from dorsal CA1 (from a subset of the tetrodes recording theta), firing rate maps (spikes/dwell time) were created in Tint (Axona), using locational bins 2.4 × 2.4 cm in size. Boxcar smoothing was applied, using 5 × 5 locational bins centered on the current bin. Spatial peak rate (spikes/dwell time, peak bin) and spatial information were calculated after smoothing, spatial information calculated in bits/spikes according to the formula in Skaggs et al. (1996). The global mean rate was calculated as total spikes/time over a whole trial.

#### 2.2.8 Analysis of theta

##### 2.2.8.1 Instantaneous theta frequency from recorded EEG

The recorded EEG signal was filtered using a 6–12 Hz, 251-tap, Blackman window erred, band-pass sinc (sine cardinal). The 6–12 Hz filtering removed non-theta-band frequencies. Windowing the filter achieves good stop-band attenuation and small pass-band ripple. A Hilbert transform was then applied, i.e., giving the analytic signal. Instantaneous theta frequency was derived by calculating theta phase differences between the adjacent time points (using 4 ms intervals). The phase of the analytic signal at a given time step gave the phase of theta at that time step and the difference in phase between each time step defined the instantaneous frequency. The rat’s position was sampled every 20 ms. The position of the rat at the time point t and t + 20 ms later enabled the calculation of instantaneous running speed. The EEG sampling rate was every 4 ms (250 Hz) and was therefore five times that of position. As such, instantaneous frequency was averaged over every five consecutive values corresponding to each position sample. In conclusion, concurrent measurements of speed and EEG theta frequency were produced every 20 ms. In this way, frequency calculation took into account the dynamic relationship between theta frequency and running speed.

##### 2.2.8.2 Theta-frequency-to-speed plots

To quantify the linear relationship between theta frequency and speed on a given trial, a regression line was calculated from the frequency-speed data points for speeds between 5 and 30 cm/s. Two variables were derived from this regression line: (a) the slope; and (b) the intercept of the regression line extended to the frequency axis at 0 cm/s.

Speeds that were below 5 cm/s were excluded in order to avoid non-theta behaviours, such as grooming. Variability of behaviour is generally highest for those behaviours when displacement is minimal. During immobility/slow speed, the rat could be doing a variety of different behaviours, whereas at higher speeds, the rat could only be walking/running. Speeds above 30 cm/s were excluded as at higher speeds theta sampling is more prone to error. Some high-speed samples are likely to represent erratic head movement and LED reflection near walls.

For the slope component of theta frequency analysis, the data from Rat 1 (first trial of Day 1) was lost. To interpolate this value, we first calculated the average of trials 2–4 for all 5 days in this rat. For days 2–5, we calculated a difference score: [(Trial 2–4 mean) – Trial 1]. We then averaged those difference scores from days 2–5, to produce a trial 1 day 1 value as follows: [(Day 1 T2–4 mean) – (Diff score D2–5, T2–4 mean = Trial 1 day 1].

#### 2.2.9 Free foraging task

Each animal received five trials (each trial 10 min long) per day over 5 days. All rats received saline injections for at least the first 3 days and received the drug injection on either the fourth (*n* = 3) or fifth (*n* = 3) day. Previous research has suggested that, on days with successive trials in the same environment, between-trial differences in both behaviour and hippocampal theta frequency are minimal across the last two trials of the day (Lever et al., 2006; Jeewajee et al., 2008b; Wells et al., 2013). Therefore, five trials per day was chosen [the equivalent in Wells et al. (2013) was four trials], with drug and saline injections performed immediately following the fourth trial. Each animal was screened daily on the holding platform for at least 2 weeks prior to the start of the experiment. On each test day, the rat was brought into the experimental room 60–75 min before the first trial of the day to enable pre-test screening and recording setup. From the holding platform, the rat was placed in the centre of the test arena for 10 min. Rats foraged for black sweetened rice. As with Wells et al. (2013), the desired latency for a drug effect (30 min) in the post-injection trial was used as the inter-trial interval for all trials (30 min), which was influenced by studies such as Siok et al. (2009) who found a theta-frequency reducing effect of pregabalin after 30 min of systemic administration in rats.

### 2.3 Open field anxiolytic drug experiment

#### 2.3.1 Animals

Subjects were male Lister Hooded rats (*n* = 29, Envigo, Wyton, UK), weighing between 315–475 g. They were housed in groups of five.

#### 2.3.2 Apparatus

External cues such as a shelf and cue cards provided directional constancy throughout. The open field (OF) arena was a grey circle (121 cm diameter, 38 cm height) suspended 20 cm above the floor. The arena was lit by ceiling lights (2,000 lux).

#### 2.3.3 Drugs and administration

Animals received a single injection of one of the following drugs: chlorodiazepoxide or CDP (2.5 mg/kg) or pregabalin (17.5 mg/kg) or saline. Injections were given 30–40 min prior to testing. The groups were assigned as follows: CDP group (*n* = 9), Pregabalin group (*n* = 10), Saline group (*n* = 10). Drugs were obtained from Sigma-Aldrich Company Ltd., Poole, UK.

#### 2.3.4 Analysis

Each animal explored the Open Field for 5 min. Ethovision 3.0 (Noldus) was used to track the movements of each animal in order to assess thigmotaxic behaviour (the tendency for the animal to remain close to the walls). A peripheral outer zone (20% of the arena’s surface) was established to calculate time spent less than 10 cm away from the walls of the arena. The frequency of rearing and grooming were manually scored using recorded footage. Rearing was defined as the animal standing on its hind-legs, whilst grooming was defined as protracted washing of the animal’s coat (Prut and Belzung, 2003).

#### 2.3.5 Open field anxiolytic task

Approximately 40-min post-injection, each animal was placed in the centre of the OF, facing the north wall, for 5 min. Behaviour was scored from recorded footage. The arena was thoroughly cleaned between animals with Azo wipes (70% IPA).

## 3 Results regarding novelty-familiarity dimension

### 3.1 The slope component of theta frequency is reduced in non-mismatch type novelty

**Figure 6:**
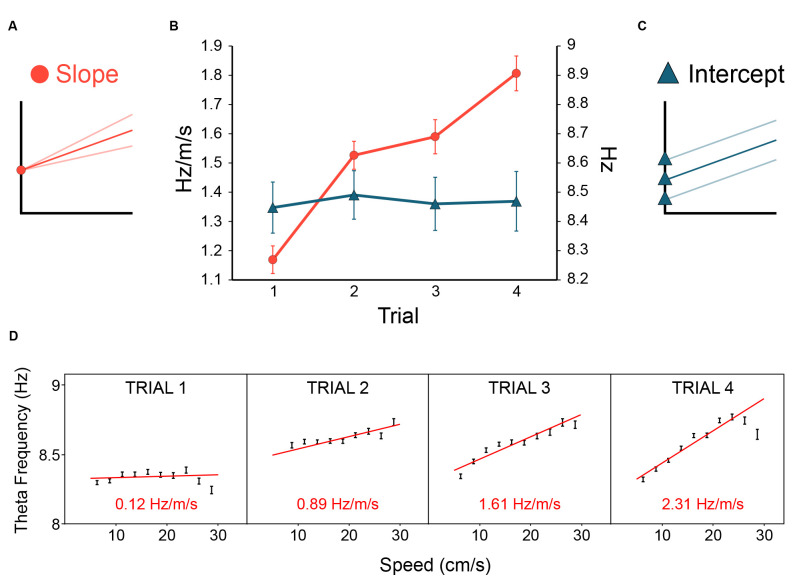
Novelty flattens theta slope, familiarity increases theta slope. In rats repeatedly exposed to the same environment (inter-trial delay : 20 min, see Methods for details), theta slope **(A)** was initially flatter on the first trial of the day, then steepened with increasing familiarity with the environment [**(B)**, red lines and circles]. In contrast, theta intercept [**(C)**, blue lines and triangles] showed no change with the novelty/familiarity dimension **(B)**. Error bars: ± 1 SEM. **(D)** A strong-effect example from a single rat over trials 1–4 on day 1 showing theta slope was initially quite flat on trial 1, and steepened over trials. Theta slope values derived from theta frequency to speed regression over 5–30 cm/s running speeds (slopes: red lines; slope values: red text, bottom; speed bins: 2.5 cm/s). In summary, novelty reduced theta frequency by selectively reducing the gain of the theta-frequency-to-stimulus-rate contribution to theta frequency.

In Section “1.7 Hippocampal theta frequency is lower in mismatch novelty and increases with familiarity” above, we asked if the novelty-elicited slope-reduction effect would generalise beyond mismatch novelty. We expected to see theta slope (Figure 6A) change according to the novelty/familiarity dimension. As predicted, Figure 6B (see red lines and circles) shows that increasing familiarisation with the environment throughout the day, from trial 1 to trial 4, resulted in theta slope increasing from trial 1 to trial 4. ANOVA^RM^ showed there was no effect of Day (*F*_(4, 20)_ = 0.248, *p* = 0.908) or Day*Trial interaction (*F*_(12, 60)_ = 0.788, *p* = 0.661), but a highly significant main effect of Trial (*F*_(3, 15)_ = 17.73, *p* < 0.0001), upon the slope. Bonferroni-adjusted pairwise comparisons showed the novelty effect, that trial 1 slopes were reliably flatter than those of other trials (*p* values ≤ 0.023; the difference between trial 1 and 2: *p* = 0.007). Importantly, as Figure 6B shows, and consistent with our previous work, this sensitivity to the novelty-familiarity dimension was only observed for theta slope and not theta intercept (Figure 6C). The equivalent ANOVA^RM^ showed there was no sign of any effects of Day, Trial, or Day*Trial interaction (*ps* ≥ 0.522) upon theta intercept. Figure 6D shows one example of theta slope steepening over trials in one rat.

### 3.2 No significant effects of novelty in dorsal CA1 place cells

Some authors have suggested increased CA1 firing rates in novelty could act as a generalised novelty signal in the hippocampus (Larkin et al., 2014). We recorded dorsal CA1 cells from a subset of the theta-recording tetrodes, to test for cellular equivalents to the effect of trial upon theta slope. We could not see any such effect (Figure 5). There was no effect of trial on *Spatial Information* [*F*_(3, 171)_ = 2.03, *p* = 0.11, (bits/spike, T1: 0.81 ± 0.07; T2: 0.89 ± 0.07; T3: 0.80 ± 0.06; T4: 0.85 ± 0.66)], *Locational Peak Rate* [*F*_(1.94, 110.4; Greenhouse-Geisser correction)_ = 5.32, *p* = 0.53, (Hz, T1: 7.92 ± 0.66; T2: 8.13 ± 0.67; T3: 8.67 ± 0.66; T4: 8.39 ± 0.66)], or *Global mean Rate* [*F*_(1.38, 78.4; G-G correction)_ = 1.87, *p* = 0.17, (Hz, T1: 1.50 ± 0.13; T2: 1.55 ± 0.14; T3: 1.74 ± 0.18; T4: 1.84 ± 0.24)]. Note that, if anything, global mean rates were *lower* in novelty. In summary, at least in our hands, theta frequency slowing is a more reliable signal of novelty than firing rate changes. Furthermore, as discussed in Section “5.7 Implications for plasticity and memory of novelty-elicits slowing of theta (NEST): the NEST hypothesis,” we see no clear pattern of firing rate changes in CA1 across different novelty-related studies.

## 4 The anxiolytic drug pregabalin selectively reduced theta intercept

In Section “3 Results regarding novelty-familiarity dimension” above, we saw the hippocampal theta slope reducing with novelty and increasing with familiarity without a change in the intercept. In Figure 1, we showed previous evidence of the converse dissociation, for hippocampal and entorhinal theta. Figure 1 pictorially summarises previous findings (hippocampal: Wells et al., 2013; entorhinal: Monaghan et al., 2017) whereby systemic injection of anxiolytic drugs reduces the intercept component, but not the slope component, of theta frequency. Intriguingly, as Gray and McNaughton have emphasised (Gray and McNaughton, 2000; McNaughton et al., 2007), theta frequency reduction by anxiolytics occurs despite the chemically different nature of the anxiolytics. We asked if this specific effect, theta intercept reduction, would also occur following the administration of pregabalin, a drug historically used to treat epilepsy and pain, but which has come to be prescribed for general anxiety in Europe. Pregabalin, which blocks α2-δ-1 subunit-containing voltage-gated calcium channels, has a different primary target to the other anxiolytic drugs tested previously (Benzodiazepines, 5HT-1A agonists, CB1 agonist). Accordingly, it provides an interesting test of our original prediction (Wells et al., 2013) that systemic anxiolytic drugs should reduce theta intercept in freely behaving rodents.

**Figure 7:**
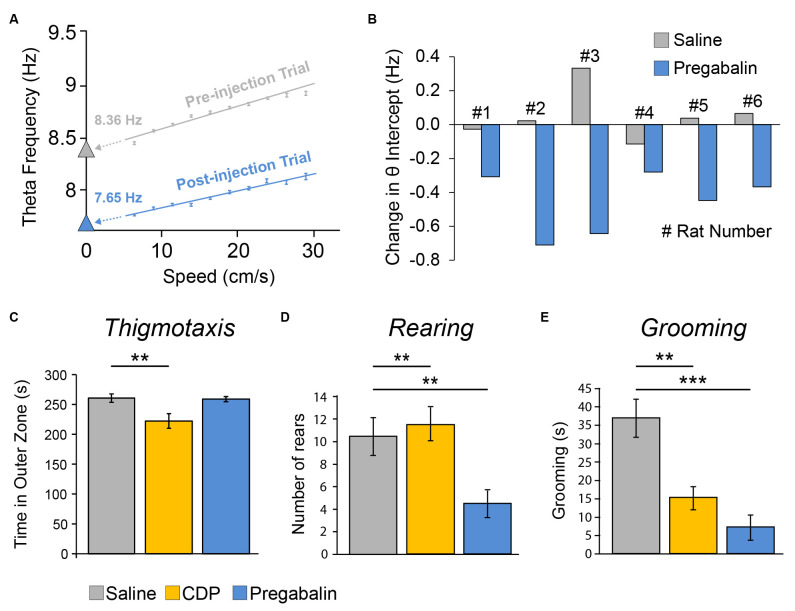
The anxiolytic drug Pregabalin reduced the theta intercept component of theta frequency (but did not elicit classic signs of anxiolysis in a separate Open Field experiment). **(A)** Single example showing theta intercept reduction by systemic injection of pregabalin. Theta frequency to speed relationship over 5–30 cm/s running speeds for pre-injection trial (grey, trial 4) and trial after higher dose (35 mg/kg, i.p.) injection of pregabalin (blue, trial 5). Theta frequency to speed regression methods as per Figure 4. The y-intercept values (theta intercept) are extrapolations of frequency-to-speed regression line extended to Y axis. **(B)** Summary data showing net theta intercept reduction following pregabalin injection, relative to saline injection, for all seven rats tested. Each bar is the difference value [Trial 4 preinjection − Trial 5 postinjection] for saline day (grey) or pregabalin day (blue) for one rat. Doses (i.p.): Rats 1–3, 35 mg/kg, Rats 4–6, 17.5 mg/kg. Pregabalin did not affect slope (data not shown). **(C–E)** In a separate experiment in a brightly lit (2,000 lux) Open Field apparatus, three groups of rats were systemically injected (i.p.) with either saline (*n* = 10), chlordiazepoxide (CDP: 2.5 mg/kg, *n* = 9), or the lower dose of pregabalinn (17.5 mg/kg, *n* = 10), Pregabalin did not elicit the classic signs of anxiolysis. **(C)** Rats given CDP did, but rats given Pregabalin did not, show reduced thigmotaxis. Pregabalin did reduce rearing relative to both saline and CDP groups **(D)**, and did reduce grooming relative to the saline group **(E)**, but these are not straightforward signs of anxiolysis. **p < 0.01, ***p < 0.001.

Exactly as predicted, pregabalin produced a net reduction in theta intercept (−0.46 ± 0.07 Hz) relative to saline injection (+0.05 ± 0.06 Hz, i.e., negligible increase) in all six animals, and this reduction was statistically significant (paired *t*_(5)_ = 4.20, *p* = 0.008; Figures 7A,B). In contrast, there was no significant effect of pregabalin on slope (*t*_(5)_ = 0.978, *p* = 0.37).

### 4.1 Pregabalin did not elicit classic signs of anxiolysis in a separate open field experiment

As a behavioural assay for anxiolytic effects of pregabalin, we tested three groups of rats injected with saline, CDP, or pregabalin in a separate open field experiment (Figures 7C–E). There was a main effect of the group upon thigmotaxis (*F*_2, 26_ = 6.21, *p* = 0.006), with *post-hoc* analysis showing that only the CDP group spent less time in the outer zone than the saline group (*p* = 0.007; Figure 7C). There was a main effect of the group upon rearing (*F*_2, 26_ = 6.63, *p* = 0.005), with post-hoc analysis showing that the pregabalin group reared less than both the saline group (*p* = 0.007) and CDP group (*p* = 0.003; Figure 7D). There was a main effect of group upon grooming (*F*_2, 26_ = 14.93, *p* = 0.00005), with post-hoc analysis showing both the pregabalin group (*p* = 0.00005) and CDP group (*p* = 0.002) groomed less than saline controls (Figure 7E). In summary, pregabalin did not reduce thigmotaxis, the classic sign of anxiety; while it did reduce rearing and grooming, the latter outcomes are not straightforward signs of anxiolysis.

## 5 Discussion

### 5.1 Summary of empirical findings

In summary, the new findings in this article support and extend our previous findings, based on spontaneous theta obtained during locomotion (Wells et al., 2013). As in Wells et al. (2013), here we observe a double dissociation in theta frequency correlates. Novelty flattens and familiarity steepens theta slope without affecting theta intercept; conversely the non-classical anxiolytic drug Pregabalin like all other anxiolytic drugs tested to date in the spontaneous locomotion theta paradigm reduces theta intercept without affecting theta slope.

### 5.2 The novelty-familiarity dimension

Regarding novelty on the first trial of the day when the environment has not been visited at all or not for 22 h theta slopes derived from locomotion-based theta are flatter. As the environment becomes more familiar and the memory of the environment is more recent 20 (min since last exposure), theta slope steepens. Thus, there appears to be a predictable relationship between the novelty-familiarity dimension and theta slope. Importantly, the general and specific (slope) reductions in theta frequency, observed in Jeewajee et al. (2008b) and Wells et al. (2013) and here, have been replicated and extended. We briefly summarise four representative studies. Regarding overall frequency: (1) Barry et al. (2012) showed that the cellular theta interburst frequency in spiking entorhinal grid cells was reduced in environmental novelty; (2) In a watermaze learning task, Hernández-Pérez et al. (2016) showed that LFP theta frequency in CA1 and CA3 was initially lower on first exposures to the watermaze, and gradually increased with increasing experience. Regarding theta slope: (3) Newman et al. (2013) observed that hippocampal LFP theta slopes (frequency-to-speed) were flatter in environmental novelty; and (4) Dannenberg et al. (2020) found that entorhinal LFP theta slopes steepen within a trial as the environment becomes familiar, extending the observation of trial-by-trial effects shown/reviewed here to smaller timescales (10 s time bins). Furthermore, as mentioned above (section “1.7 Hippocampal theta frequency is lower in mismatch novelty and increases with familiarity”), object-related novelty also elicits hippocampal theta frequency reduction. Taking all these studies together, then, it seems to be a general property of novelty that it elicits theta frequency reduction, particularly in the stimulus-rate contribution to theta frequency. This property is perhaps not widely known, despite the special status of novelty for learning, and so we consider further implications of this in Section “5.7 Implications for plasticity and memory of novelty-elicits slowing of theta (NEST): the NEST hypothesis” below.

### 5.3 Anxiolytic drug action

Consistent with the theoretical framework outlined in Wells et al. (2013), based on Burgess (2008), we found here that the anxiolytic drug pregabalin reduces the theta intercept component of theta frequency. It is interesting that the specific effect of y-intercept reduction in hippocampal formation theta from the freely moving rat is shared by all these anxiolytic drugs tested (Wells et al., 2013; Monaghan et al., 2017; this study). This common specific effect occurs despite both: (a) the considerable variance in primary targets (Figure 1H); and (b) the presynaptic and postsynaptic locations of these targets. For example: (a) chlordiazepoxide (Wells et al., 2013) and diazepam (Monaghan et al., 2017) activate the benzodiazepine site of the GABA-A receptors, buspirone (Wells et al., 2013) and 8-OH-DPAT (Monaghan et al., 2017) activate 5HT-1A receptors, and pregabalin (this study) activates the α2-δ-1 subunit-containing voltage-gated calcium channel; and (b) the benzodiazepine site of the GABA-A receptor is postsynaptic, while the α2-δ-1 subunit-containing voltage-gated calcium channel is presynaptic.

### 5.4 Theta intercept reduction as an assay for anxiolytic drug action?

We note, consistent with theory-based claims in McNaughton et al. (2007), but extended to theta intercept based on spontaneous locomotion, that our theta-frequency reduction assay “predicts” the anxiolytic efficacy of pregabalin in humans. In contrast, an open-field behavioural assay did not (i.e., no sign of Pregabalin-reduced thigmotaxis), at least not conventionally, since while Pregabalin did reduce rearing and grooming, neither outcome is a straightforward sign of anxiolysis. This suggests that theta-intercept reduction in the spontaneously moving rodent may function as a more predictive assay of the anxiolytic efficacy of candidate anxiolytic drugs in humans than a classic rodent behavioural assay of anxiolysis. Even if theta frequency reduction *per se* is not the critical factor in anxiolysis, the potential to examine the correlates of theta-intercept reduction in locomotion-based theta during free behaviour may provide a clue to fundamental anxiolytic mechanisms. Importantly, the common effect of hippocampal theta frequency reduction by systemically-administered anxiolytic drugs across widely-different drug types in both the spontaneous-locomotion theta approach and the reticular-stimulation theta approach provide strong support for a “final common pathway” in anxiolysis, involving the hippocampal formation (Gray and McNaughton, 2000).

### 5.5 Theta slope: robustness and generality of the theta frequency to stimulus rate relationship?

The account we have presented here takes the positive correlation between theta frequency and locomotion speed as a general model of theta frequency to stimulus rate. How robust is this model? We note that the theta-frequency-speed correlation has been observed in many tens of rodent experiments, in both the local field potential (LFP) and the interburst-frequency of hippocampal formation neurons (reviewed Korotkova et al., 2018; see Hinman et al., 2016; Dannenberg et al., 2020; Kennedy et al., 2022 for updates and mechanistic insights), and recently in human hippocampal LFP theta in a visual VR task (Goyal et al., 2020). Although there has been a single, recent report that theta frequency correlates with acceleration and *not* speed (Kropff et al., 2021), this claim has been carefully investigated, including a re-analysis of the dataset, and not been supported (Kennedy et al., 2022).

Is the frequency-speed relationship in rodents a representative model of a more general frequency-to-stimulus-rate relationship? There is currently little data to speak to this question. Interestingly, a recent study (Santos-Pata et al., 2022) examined human hippocampal theta frequency in a purely visually-based VR task where subjects experience passive motion on a circular track. Speed and the number of cues, which were equally spaced over the track, were constant throughout a trial, but varied independently across trials in a within-subject design. They found that hippocampal theta frequency positively correlated with cue rate more than speed (Santos-Pata et al., 2022). The relative influence deserves further exploration since many typically-important self-motion cues to locomotion speed such as from vestibular, proprioceptive, and motor efferent copy systems (reviewed Poulter et al., 2018) are of course unavailable in visual VR tasks. However, it is intriguing that the number of distinct visual cues presented per second appears to be an isolatable factor positively correlating with theta frequency. Studies such as Goyal et al. (2020) and Santos-Pata et al. (2022) encouragingly suggest that theta slope and theta intercept may become tractable variables for study in humans (with intracranial recordings).

### 5.6 Future experiments regarding timing

The tractability of theta slope and intercept variables offers scope for across-species experiments in timing. We suggested above that one route to progress in evaluating novelty effects upon timing is to induce a more tonic novelty state, such as being placed in an obviously new, but non-anxiogenic, context for some time. Once a timing-related paradigm is set up with time cells and theta variables recorded in a standard configuration, the effect of variables such as novelty could be systematically tested. For instance, the spatial context could be changed around the same apparatus to elicit a novelty response. We would predict that, in initial trials in novelty, the temporal width of hippocampal time cell fields would be expanded and theta phase precession slopes would be flatter as if time was “stretching out.” It follows, albeit with less empirical foundation, that we would also predict the same kind of result under lower brain temperatures, and conversely smaller temporal fields and steeper precession slopes for higher brain temperatures, relative to the baseline condition. In peak responding experiments, we would predict that, relative to baseline conditions, higher brain temperatures would result in later, and lower brain temperatures in earlier, peak responding. It remains an entirely open question as to what extent changes in intercept relate to timing.

### 5.7 Implications for plasticity and memory of novelty-elicits slowing of theta (NEST): the NEST hypothesis

What are the implications for plasticity and memory that theta frequency is reduced in novelty? As we have noted above (Sections “1.7 Hippocampal theta frequency is lower in mismatch novelty and increases with familiarity” and “5.2 The novelty-familiarity dimension”), the findings in rodents on theta frequency slowing during novelty and speeding up during familiarization, and this effect primarily being due to theta slope variation, have been well replicated. Considering the strong relationship between learning and novelty, the set of empirical findings showing theta frequency slowing in novelty calls for speculation as to its implications for memory. A consensus feature of conceptions of novel situations is that they entail adaptive new learning. In terms of spatial novelty, in “uncharted novelty,” a new model should be built; in “mismatch novelty,” an existing model should be updated (O’Keefe and Nadel, 1978; Hasselmo et al., 1996; Lever et al., 2006), and so on. Such learning requires enhanced plasticity (Lisman and Grace, 2005). While a lot remains unknown about learning and storage mechanisms, synaptic plasticity is likely to be important in novelty-elicited coding. Interestingly, then, both the sign (e.g., depression, potentiation) and the magnitude (e.g., short-term, long-term) of synaptic plasticity are modulated by the theta cycle (reviewed in Hasselmo et al., 2002; Hasselmo, 2011; Korotkova et al., 2018). For instance, for CA1 pyramidal layer theta, theta-peak timed stimulation is thought to be most beneficial for LTP.

In summary, then, a new hypothesis attempting to incorporate theoretical and empirical ideas on novelty, theta, and memory is called for. Accordingly, here we set out key elements in our Novelty-Elicits Slowing of Theta (NEST) hypothesis as follows.

**Figure 8:**
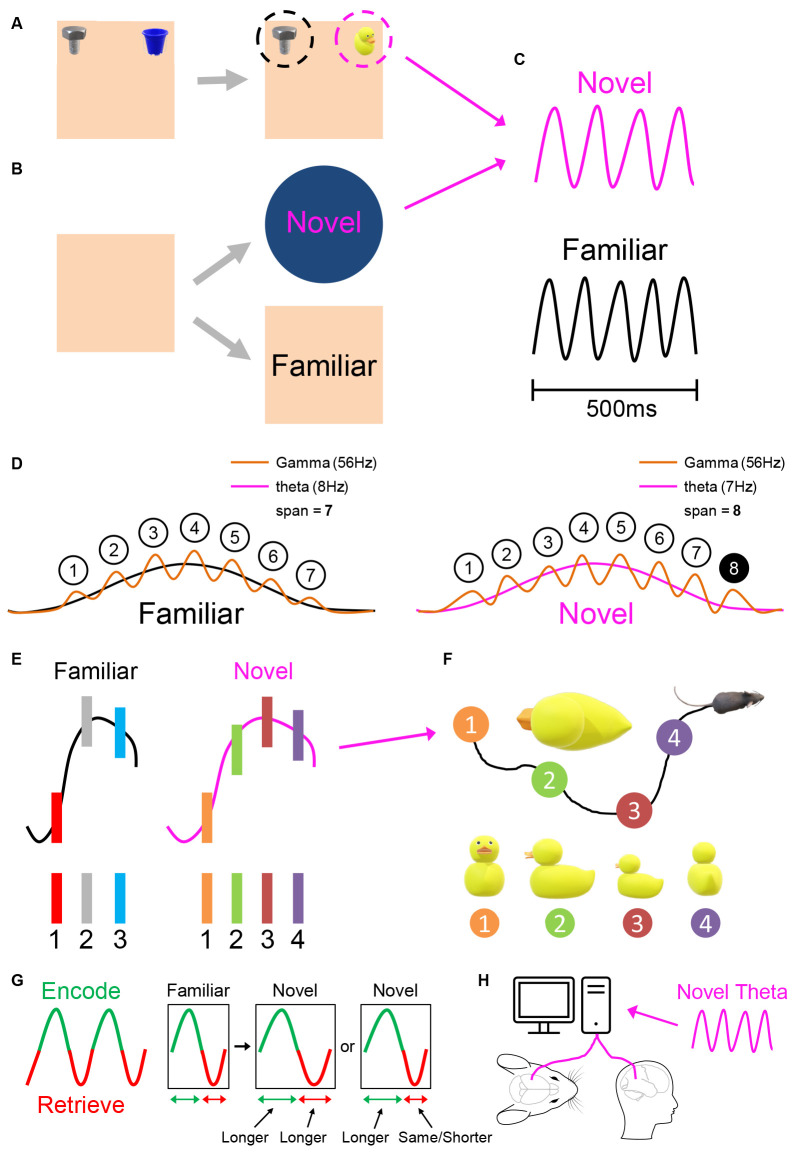
Elements of the Novelty-Elicits Slowing of Theta frequency (NEST) hypothesis. When exposed to novelty, such as to a novel object-in-place configuration **(A)**, or novel spatial context **(B)**, hippocampal theta frequency slows, and thus cycles lengthen **(C)**. The NEST hypothesis proposes that such slowing is a general hippocampal-wide response to novelty, to enhance the additional need for associativity (quantity and memorability of information) that novelty calls for. This enhancement is a general principle, not necessarily tied to theta-gamma coupling, though some existing theory and data regarding theta-gamma coupling suit this principle **(D,E)**. In Lisman and Idiart (1995), modelling the presumed 7 ± 2 item capacity (“span”) of working memory (WM), each gamma cycle is associated with one to-be-remembered item, and thus memory span corresponds to the total number of gamma cycles nested within one theta cycle **(D)**. In one model in Jensen and Lisman (1998), theta frequency is adaptable. If gamma frequency is constant across familiar vs. novel conditions, but theta frequency slows in novelty (right), then WM span increases in the novel condition, here from 7–8 items. In models of longer-term memory, e.g., Hasselmo et al. (2002), theta phase predicts both the sign and strength of synaptic plasticity, but the same principle applies that longer cycles would enable more within-cycle memoranda within the same phase-distance envelope, e.g., here three items in the familiar condition (**E**, left), and four items in the novel condition (**E**, right). The temporal contingencies of theta sequences suit spike-timing-dependent plasticity, and boosted by neuromodulation and subsequent “replay,” longer cycles enable more items such as successive locations (views) around (of) a single object **(F)**, to be bound together in long-term memory. Not all the theta cycle may need to lengthen. If a specific portion of theta cycle suits encoding **(G)**, (Hasselmo et al., 2002), such as peak-half (example shown here, green) or ascending half (not shown), cycle lengthening need only occur for the encoding-relevant portion of the cycle. Finally, if the NEST hypothesis has merit, then artificial stimulation aiming at boosting memory encoding should use novelty-like slower frequencies **(H)**. See main text for further discussion. Part **(D)** adapted from Jensen and Lisman (1998) and Vosskuhl et al. (2015).

(1) **Theta frequency slowing in the hippocampal formation is a generalised response to novelty of different types (e.g., uncharted vs. mismatch) and modalities (e.g., spatial, objects, peers, predators).** See Figures 8A,B. Thus we predict that this rodent-based NEST finding will extend to other mammalian species, and that the degree of theta slowing is proportional to the degree of novelty.
(2) Novelty-elicited theta slowing is not an epiphenomenon, but an adaptive hippocampal-formation-wide response functioning to accommodate the additional need for learning entailed by novelty. Changes in hippocampal theta power and firing rate seem to be much less consistent responses to novelty than theta slowing. *Power*: e.g., in Wells et al. (2013) speed-controlled theta power significantly increased in one novelty experiment (see also Penley et al., 2013), but not in another (Human data also suggests no consistency, e.g., increase: Chen et al., 2013; decrease: Kaplan et al., 2012; Park et al., 2014). *Firing rate*: even when restricted to a single region (rodent CA1 place/pyramidal cells), all possible results are seen: *increases* in novelty e.g., Nitz and McNaughton (2004), Csicsvari et al. (2007), and Larkin et al. (2014); *decreases* in novelty e.g., Frank et al. (2004), Kitanishi et al. (2015), and Pfeiffer (2022); and unreliable changes in either direction, e.g., (increase: Hirase et al., 2001; decrease: Douchamps et al., 2013; this study). Thus we posit that the most common and generalised adaptive response is theta slowing. Since this presumptive signal occurs throughout the hippocampal formation, including the dentate gyrus, Cornu Ammonis regions, subiculum, and entorhinal cortex (Jeewajee et al., 2008b; Wells et al., 2013; Section “5.2 The novelty-familiarity dimension” above), frequency reduction can coincide with communication requiring frequency-based theta coherence across regions within the hippocampal formation (e.g., Gray and McNaughton, 2000; Fries, 2005).
(3) **Lengthening the theta cycle enhances associativity.** See Figures 8D–F. This enhancement extends to the quantity and memorability of information, i.e., to the number and/or sequence of memoranda, and, in concert with neuromodulation, to how well information is remembered over the short and long term. We presume this enhancement is a general principle, and is not necessarily tied to theta-gamma coupling, though already-existing theory (Lisman and Idiart, 1995; Jensen and Lisman, 2005) and data (discussed below) on theta-gamma coupling suit this principle. In a study of mismatch novelty (new floor in same spatial context), the task epoch in which novelty-elicited hippocampal theta cycles were longer than the familiar condition coincided with increased theta coherence and increased theta-gamma coupling (both CA3- and Entorhinal-linked gamma; López-Madrona et al., 2020). Thus the NEST phenomenon may dovetail with increases in theta-gamma coupling in novelty.
(4) **Even part-cycle lengthening may boost associativity.** See Figure 8G. *How much* of the whole theta cycle needs to be lengthened, in what circumstances, is an interesting question empirically. In the frameworks of Lisman and colleagues, in which 7 ± 2 gamma cycles nest within one theta cycle, and each gamma cycle corresponds to one item, about $\frac{3}{4}$ of the theta cycle is available for association, e.g., Jensen and Lisman (1998), with “dead time” near the theta trough. In the simplest reading of the Hasselmo et al. (2002) model and related studies (see Section “1.3 Quantal time: the theta cycle as a cognitive moment?” above), only a half-cycle is available and beneficial for encoding. Amemiya and Redish (2018) examined Vicarious-Trial-&-Error (VTE) behaviour, which has similarities with novelty-driven behaviour (e.g., increased attention to the sensory world), and noted a half-cycle related change. The general observation was that slower theta frequencies occurred in VTE-containing trials before and after the maze choice point. Intriguingly, closer inspection revealed that this was largely driven by the lengthening of one half-cycle, the ascending phase (fissure-referenced theta) associated with low-gamma. Thus, there is likely an adaptive modifiability with respect to which fractions of the theta cycle change and how much by. The current working assumption based on CA1 is that particular gamma bands are strongly tied to theta phase (Colgin et al., 2009; Belluscio et al., 2012; Scheffer-Teixeira et al., 2012; Schomburg et al., 2014; Lopes-dos-Santos et al., 2018), and thus define the encoding-oriented (Entorhinal-driven mid-gamma) vs. retrieval-oriented (CA3-driven slow-gamma) theta phases (reviewed Hasselmo, 2011; Easton et al., 2012; Colgin, 2013). However, theta-gamma relationships are likely more complex than previously thought (Lopes-dos-Santos et al., 2018), and are often characterised when tasks are well-learned. Intriguingly, Rayan et al. (2022) found that the theta-phase preference of slow-gamma and mid/fast-power shifted with learning in the starting box of a spatial reference memory task. Remarkably, the theta-phase preference of slow-gamma shifted 140° as rats progressed from “novice” to “skilled” learners in a spatial reference memory task. Thus theta-gamma relationships may be more plastic than previously thought, and slow-gamma phases involved with learning.
(5) **Artificial theta stimulation aimed at enhancing learning should employ low-end theta frequencies.** See Figure 8H. Generally, if the NEST hypothesis has merit, it suggests that increasing packet size is more beneficial for learning in the trade-off between information packet size (longer cycles preferred) and repetitions of the information packet (more cycles preferred). Thus, in addition to considering the appropriate *phase* of theta for enhancing *encoding* vs. retrieval (Siegle and Wilson, 2014), novelty-like frequencies at the lower end of the theta frequency band should be considered for stimulation aimed at enhancing encoding.

Of course, the processes entailed here in the NEST hypothesis do not complete the plasticity story. For instance, information embedded in theta phase precession and theta sequences likely need replay for longer-term storage (Drieu et al., 2018). Finally, novelty-elicited slowing of theta is one of several novelty-related processes linked to memory, thus the NEST hypothesis posits a gain-of-function from cycle-lengthening other things being equal. Most obviously, if amnestic (e.g., drug-induced) processes coincide with theta frequency slowing (e.g., reviewed Gray and McNaughton, 2000), then theta frequency slowing by itself is not sufficient to boost associativity.

Our literature review above in Sections “1.7 Hippocampal theta frequency is lower in mismatch novelty and increases with familiarity” and “5.2 The novelty-familiarity dimension” presented empirical support for the initial end of the process proposed in the NEST hypothesis, that theta cycles lengthen in novelty. We now turn to the more speculative part of the concept, that theta cycle lengthening boosts associative memory. Firstly, the NEST hypothesis has some indirect support from manipulations of working memory load in humans. Intracranial recordings of human hippocampal theta show that theta frequency decreases, i.e., theta cycles lengthen, as the number of to-be-remembered items increases (Axmacher et al., 2010). This occurs alongside increased theta-gamma coupling, consistent with the idea that lengthened cycles enable more gamma-cycle units of representation to be nested within a single theta cycle (Jensen and Lisman, 1998; Axmacher et al., 2010). Similarly, using MEG in a visual working memory task, Moran et al. (2010) found that theta oscillations moved to lower frequencies in a hippocampal-centric network including the posterior hippocampus and occipital cortex, to support successful memory for higher loads. Building upon such work, Wolinski et al. (2018; see also Vosskuhl et al., 2015), investigated the causal role of theta frequency variation using transcranial alternating current stimulation (tACS) targeting the intraparietal sulcus. The key within-subject effect was as follows. Relative to sham stimulation, and only for the visual hemifield contralateral to the stimulation site, slower (4 Hz tACS) theta frequency stimulation *increased*, and faster (7 Hz tACS) theta frequency stimulation *decreased*, working memory capacity for visual items (Wolinski et al., 2018).

We turn, secondly, to rodent work and longer-term memory. Trimper et al. (2014) looked at rats’ ability to remember objects-in-place for 0.5–2 min intervals. They found that the portion of the hippocampal theta cycle which showed high CA3-CA1 low-gamma coherence was *elongated* at encoding for subsequently better-remembered objects. Regarding promnestic stimulation, a promising hint was provided by Quirk et al. (2021) using optogenetic stimulation of PV+ medial septal cells. They reported amnestic effects of higher-theta frequency stimulation (≥10 Hz) when applied during encoding-relevant (but not retrieval-relevant) maze segments of a spatial alternation task. At the longer, 10-second, interval which produced the strongest amnestic effects from high theta-frequency stimulation (12 Hz), there was a trend towards better memory at slower frequencies (4 and 6 Hz, but where sample sizes were smaller; Extended Data Figure 8, Supplementary Data 3, in Quirk et al., 2021). To our knowledge, other studies have not systematically compared different frequencies of stimulation. In two studies where theta-frequency stimulation replaced (McNaughton et al., 2006) or competed with (Mouchati et al., 2020) endogenous theta, and spatial learning was relatively intact, low-middle theta frequencies were employed (7.7 Hz, McNaughton et al., 2006; 6 Hz, Mouchati et al., 2020.

In summary, there is some support for adaptive change in theta cycle length, linked in turn to enhancing associativity, and even hints that low theta frequency may be most beneficial for promnestic stimulation therapies. This is clearly an area in which empirical findings are greatly to be welcomed, and we hope that the NEST hypothesis is a useful spur to such work.

### 5.8 Conclusions

Attention should be paid to frequency as well as to other variables relating to hippocampal theta. While the relationship between hippocampal theta frequency and locomotion speed is widely appreciated, the influence of other factors such as novelty and anxiolytic drugs are neglected. This neglect occurs despite apparently good evidence, reviewed here, for doubly dissociable effects of novelty upon theta slope, and anxiolytic drugs upon theta intercept, as defined from locomotion-derived theta in freely behaving animals. These hippocampal-wide changes correlating with cognitive and emotional variables are likely to provide important clues to function, such as subjective time, learning, and generalised anxiety. In particular, the evidence linking novelty to theta frequency reduction, broadly defined, is accumulating across different labs, and studying this further may help understand hippocampal encoding processes. Species differences in hippocampal theta do occur across different mammalian species. For instance, compared to rodents, theta is more prevalent in the ferret (Dunn et al., 2022), and less so in humans; however more insight is emerging into human theta *via* intracranial recordings and MEG. We anticipate such studies will likely reveal broadly similar theta-slope “stimulus rate” frequency correlates (Santos-Pata et al., 2022), and theta-intercept “baseline” frequency correlates, as obtained in locomoting rodents. We predict that novelty-elicited theta slowing occurs in several mammalian species, and forms part of a general “need for additional learning” adaptive process.

## Data availability statement

Data are available upon reasonable request from the authors.

## Ethics statement

All experiments were performed under the Animals (Scientific Procedures) Act 1986. Approval for the animal experiments was granted by both Durham University AWERB and the UK Home Office Project and Personal Licenses.

## Author contributions

All authors contributed to the article and approved the submitted version.

## References

[B1] Amemiya S. Redish A. D. (2018). Hippocampal theta-gamma coupling reflects state-dependent information processing in decision making. Cell Rep. 22, 3328–3338. 10.1016/j.celrep.2018.02.091 29562187 PMC5929482

[B2] Aschoff J. (1998). Human perception of short and long time intervals: its correlation with body temperature and the duration of wake time. J. Biol. Rhythms 13, 437–442. 10.1177/074873098129000264 9783235

[B3] Axmacher N. Henseler M. M. Jensen O. Weinreich I. Elger C. E. Fell J. (2010). Cross-frequency coupling supports multi-item working memory in the human hippocampus. Proc. Natl. Acad. Sci. U S A 107, 3228–3233. 10.1073/pnas.0911531107 20133762 PMC2840289

[B4] Baddeley A. D. (1966). Time-estimation at reduced body-temperature. Am. J. Psychol. 79, 475–479. 5968486

[B5] Bannerman D. M. Rawlins J. N. P. McHugh S. B. Deacon R. M. J. Yee B. K. Bast T.. (2004). Regional dissociations within the hippocampus—memory and anxiety. Neurosci. Biobehav. Rev. 28, 273–283. 10.1016/j.neubiorev.2004.03.004 15225971

[B6] Barnett A. J. O’Neil E. B. Watson H. C. Lee A. C. (2014). The human hippocampus is sensitive to the durations of events and intervals within a sequence. Neuropsychologia 64, 1–12. 10.1016/j.neuropsychologia.2014.09.011 25223466

[B7] Barry C. Ginzberg L. L. O’Keefe J. Burgess N. (2012). Grid cell firing patterns signal environmental novelty by expansion. Proc. Natl. Acad. Sci. U S A 109, 17687–17692. 10.1073/pnas.1209918109 23045662 PMC3491492

[B514] Barth A. M. Domonkos A. Fernandez-Ruiz A. Freund T. F. Varga V. (2018). Hippocampal network dynamics during rearing episodes. Cell Rep. 23, 1706–1715. 10.1016/j.celrep.2018.04.021 29742427 PMC5978794

[B8] Belluscio M. A. Mizuseki K. Schmidt R. Kempter R. Buzsáki G. (2012). Cross-frequency phase-phase coupling between theta and gamma oscillations in the hippocampus. J. Neurosci. 32, 423–435. 10.1523/JNEUROSCI.4122-11.2012 22238079 PMC3293373

[B9] Brandon M. P. Bogaard A. R. Schultheiss N. W. Hasselmo M. E. (2013). Segregation of cortical head direction cell assemblies on alternating theta cycles. Nat. Neurosci. 16, 739–748. 10.1038/nn.3383 23603709 PMC3703458

[B10] Buhusi C. V. Meck W. H. (2005). What makes us tick? Functional and neural mechanisms of interval timing. Nat. Rev. Neurosci. 6, 755–765. 10.1038/nrn1764 16163383

[B502] Buhusi C. V. Mocanu M. Meck W. H. (2004). Abnormal memory consolidation of interval timing in rats with ibotenic lesions of the hippocampus. Soc. Neurosci. 34:550.18.

[B11] Burgess N. (2008). Grid cells and theta as oscillatory interference: theory and predictions. Hippocampus 18, 1157–1174. 10.1002/hipo.20518 19021256 PMC3196519

[B12] Burgess N. Barry C. O’Keefe J. (2007). An oscillatory interference model of grid cell firing. Hippocampus 17, 801–812. 10.1002/hipo.20327 17598147 PMC2678278

[B13] Chen J. Dastjerdi M. Foster B. L. LaRocque K. F. Rauschecker A. M. Parvizi J.. (2013). Human hippocampal increases in low-frequency power during associative prediction violations. Neuropsychologia 51, 2344–2351. 10.1016/j.neuropsychologia.2013.03.019 23571081 PMC3805697

[B515] Clarke J. Porubanova M. (2020). Scene and object violations cause subjective time dilation. Timing Time Percept. 8, 279–298. 10.1163/22134468-bja10012

[B506] Climer J. R. Newman E. L. Hasselmo M. E. (2013). Phase coding by grid cells in unconstrained environments: two-dimensional phase precession. Eur. J. Neurosci. 38, 2526–2541. 10.1111/ejn.12256 23718553 PMC3912569

[B14] Colgin L. L. (2013). Mechanisms and functions of theta rhythms. Annu. Rev. Neurosci. 36, 295–312. 10.1146/annurev-neuro-062012-170330 23724998

[B15] Colgin L. L. Denninger T. Fyhn M. Hafting T. Bonnevie T. Jensen O.. (2009). Frequency of gamma oscillations routes flow of information in the hippocampus. Nature 462, 353–357. 10.1038/nature08573 19924214

[B16] Csicsvari J. O’Neill J. Allen K. Senior T. (2007). Place-selective firing contributes to the reverse-order reactivation of CA1 pyramidal cells during sharp waves in open-field exploration. Eur. J. Neurosci. 26, 704–716. 10.1111/j.1460-9568.2007.05684.x 17651429 PMC2121123

[B17] Cutsuridis V. Hasselmo M. (2012). GABAergic contributions to gating, timing and phase precession of hippocampal neuronal activity during theta oscillations. Hippocampus 22, 1597–1621. 10.1002/hipo.21002 22252986

[B18] Dannenberg H. Lazaro H. Nambiar P. Hoyland A. Hasselmo M. E. (2020). Effects of visual inputs on neural dynamics for coding of location and running speed in medial entorhinal cortex. eLife 9:e62500. 10.7554/eLife.62500 33300873 PMC7773338

[B510] Deboer T. (2002). Electroencephalogram theta frequency changes in parallel with euthermic brain temperature. Brain Res. 930, 212–215. 10.1016/s0006-8993(02)02247-3 11879812

[B511] Deboer T. Tobler I. (1995). Temperature dependence of EEG frequencies during natural hypothermia. Brain Res. 670, 153–156. 10.1016/0006-8993(94)01299-w 7719716

[B19] Douchamps V. Jeewajee A. Blundell P. Burgess N. Lever C. (2013). Evidence for encoding versus retrieval scheduling in the hippocampus by theta phase and acetylcholine. J. Neurosci. 33, 8689–8704. 10.1523/JNEUROSCI.4483-12.2013 23678113 PMC3715394

[B20] Drieu C. Todorova R. Zugaro M. (2018). Nested sequences of hippocampal assemblies during behavior support subsequent sleep replay. Science 362, 675–679. 10.1126/science.aat2952 30409880

[B21] Dunn S. L. S. Town S. M. Bizley J. K. Bendor D. (2022). Behaviourally modulated hippocampal theta oscillations in the ferret persist during both locomotion and immobility. Nature Commun. 13:5905. 10.1038/s41467-022-33507-2 36207304 PMC9547075

[B22] Easton A. Douchamps V. Eacott M. Lever C. (2012). A specific role for septohippocampal acetylcholine in memory?. Neuropsychologia 50, 3156–3168. 10.1016/j.neuropsychologia.2012.07.022 22884957 PMC3605586

[B23] Engin E. Stellbrink J. Treit D. Dickson C. T. (2008). Anxiolytic and antidepressant effects of intracerebroventricularly administered somatostatin: behavioural and neurophysiological evidence. Neuroscience 157, 666–676. 10.1016/j.neuroscience.2008.09.037 18940236

[B24] Field M. J. Oles R. J. Singh L. (2001). Pregabalin may represent a novel class of anxiolytic agents with a broad spectrum of activity. Br. J. Pharmacol. 132, 1–4. 10.1038/sj.bjp.0703794 11156553 PMC1572552

[B25] Frank L. M. Stanley G. B. Brown E. N. (2004). Hippocampal plasticity across multiple days of exposure to novel environments. J. Neurosci. 24, 7681–7689. 10.1523/JNEUROSCI.1958-04.2004 15342735 PMC6729632

[B26] Fries P. (2005). A mechanism for cognitive dynamics: neuronal communication through neuronal coherence. Trends Cogn. Sci. 9, 474–480. 10.1016/j.tics.2005.08.011 16150631

[B27] Gambeta E. Kopruszinski C. M. Dos Reis R. C. Zanoveli J. M. Chichorro J. G. (2017). Facial pain and anxiety-like behavior are reduced by pregabalin in a model of facial carcinoma in rats. Neuropharmacology 125, 263–271. 10.1016/j.neuropharm.2017.07.035 28778832

[B28] Goyal A. Miller J. Qasim S. E. Watrous A. J. Zhang H. Stein J. M.. (2020). Functionally distinct high and low theta oscillations in the human hippocampus. Nat. Commun. 11:2469. 10.1038/s41467-020-15670-6 32424312 PMC7235253

[B30] Gray J. A. (1982). Précis of the neuropsychology of anxiety: an enquiry into the functions of the septo-hippocampal system. Behav. Brain Sci. 5, 469–484.

[B29] Gray J. A. McNaughton N. (2000). The Neuropsychology of Anxiety. London: Oxford University Press.

[B31] Gu B. M. van Rijn H. Meck W. H. (2015). Oscillatory multiplexing of neural population codes for interval timing and working memory. Neurosci. Biobehav. Rev. 48, 160–185. 10.1016/j.neubiorev.2014.10.008 25454354

[B33] Hancock P. A. (1993). Body temperature influence on time perception. J. Gen. Psychol. 120, 197–216. 10.1080/00221309.1993.9711144 8138792

[B34] Harter M. R. (1967). Excitability cycles and cortical scanning: a review of two hypotheses of central intermittency in perception. Psychol. Bull. 68, 47–58. 10.1037/h0024725 4859873

[B35] Hasselmo M. E. (2011). How We Remember: Brain Mechanisms of Episodic Memory. London: MIT press.

[B36] Hasselmo M. E. Bodelón C. Wyble B. P. (2002). A proposed function for hippocampal theta rhythm: separate phases of encoding and retrieval enhance reversal of prior learning. Neural Comput. 14, 793–817. 10.1162/089976602317318965 11936962

[B509] Hasselmo M. E. Stern C. E. (2014). Theta rhythm and the encoding and retrieval of space and time. Neuroimage 85, 656–666. 10.1016/j.neuroimage.2013.06.022 23774394 PMC3918488

[B37] Hasselmo M. E. Wyble B. P. Wallenstein G. V. (1996). Encoding and retrieval of episodic memories: role of cholinergic and GABAergic modulation in the hippocampus. Hippocampus 6, 693–708. 10.1002/(SICI)1098-1063(1996)6:6<693::AID-HIPO12>3.0.CO;2-W 9034856

[B38] Hernández-Pérez J. J. Gutiérrez-Guzmán B. E. Olvera-Cortés M. E. (2016). Hippocampal strata theta oscillations change their frequency and coupling during spatial learning. Neuroscience 337, 224–241. 10.1016/j.neuroscience.2016.09.003 27615031

[B40] Hinman J. R. Brandon M. P. Climer J. R. Chapman G. W. Hasselmo M. E. (2016). Multiple running speed signals in medial entorhinal cortex. Neuron 91, 666–679. 10.1016/j.neuron.2016.06.027 27427460 PMC4976037

[B41] Hirase H. Leinekugel X. Czurkó A. Csicsvari J. Buzsáki G. (2001). Firing rates of hippocampal neurons are preserved during subsequent sleep episodes and modified by novel awake experience. Proc. Natl. Acad. Sci. U S A 98, 9386–9390. 10.1073/pnas.161274398 11470910 PMC55430

[B42] Hoagland H. (1933). The physiological control of judgments of duration: evidence for a chemical clock. J. Gen. Psychol. 9, 267–287. 10.1080/00221309.1933.9920937

[B505] Huxter J. R. Senior T. J. Allen K. Csicsvari J. (2008). Theta phase–specific codes for two-dimensional position, trajectory and heading in the hippocampus. Nat. Neurosci. 11, 587–594. 10.1038/nn.2106 18425124

[B43] Hyman J. M. Wyble B. P. Goyal V. Rossi C. A. Hasselmo M. E. (2003). Stimulation in hippocampal region CA1 in behaving rats yields long-term potentiation when delivered to the peak of theta and long-term depression when delivered to the trough. J. Neurosci. 23, 11725–11731. 10.1523/JNEUROSCI.23-37-11725.2003 14684874 PMC6740943

[B44] Itskov V. Curto C. Pastalkova E. Buzsáki G. (2011). Cell assembly sequences arising from spike threshold adaptation keep track of time in the hippocampus. J. Neurosci. 31, 2828–2834. 10.1523/JNEUROSCI.3773-10.2011 21414904 PMC3097063

[B45] Jeewajee A. Barry C. O’Keefe J. Burgess N. (2008a). Grid cells and theta as oscillatory interference: electrophysiological data from freely moving rats. Hippocampus 18, 1175–1185. 10.1002/hipo.20510 19021251 PMC3173868

[B46] Jeewajee A. Lever C. Burton S. O’keefe J. Burgess N. (2008b). Environmental novelty is signaled by reduction of the hippocampal theta frequency. Hippocampus 18, 340–348. 10.1002/hipo.20394 18081172 PMC2678674

[B507] Jeewajee A. Barry C. Douchamps V. Manson D. Lever C. Burgess N. (2014). Theta phase precession of grid and place cell firing in open environments. Philos. Trans. R Soc. B Biol. Sci. 369:20120532. 10.1098/rstb.2012.0532 24366140 PMC3866450

[B47] Jensen O. Lisman J. E. (1998). An oscillatory short-term memory buffer model can account for data on the Sternberg task. J. Neurosci. 18, 10688–10699. 10.1523/JNEUROSCI.18-24-10688.1998 9852604 PMC6793327

[B48] Jensen O. Lisman J. E. (2005). Hippocampal sequence-encoding driven by a cortical multi-item working memory buffer. Trends Neurosci. 28, 67–72. 10.1016/j.tins.2004.12.001 15667928

[B49] Jezek K. Henriksen E. J. Treves A. Moser E. I. Moser M. B. (2011). Theta-paced flickering between place-cell maps in the hippocampus. Nature 478, 246–249. 10.1038/nature10439 21964339

[B50] Kaplan R. Doeller C. F. Barnes G. R. Litvak V. Düzel E. Bandettini P. A.. (2012). Movement-related theta rhythm in humans: coordinating self-directed hippocampal learning. PLoS Biol. 10:e1001267. 10.1371/journal.pbio.1001267 22389627 PMC3289589

[B51] Kay K. Chung J. E. Sosa M. Schor J. S. Karlsson M. P. Larkin M. C.. (2020). Constant sub-second cycling between representations of possible futures in the hippocampus. Cell 180, 552–567.e25. 10.1016/j.cell.2020.01.014 32004462 PMC7126188

[B52] Kennedy J. P. Zhou Y. Qin Y. Lovett S. D. Sheremet A. Burke S. N.. (2022). A direct comparison of theta power and frequency to speed and acceleration. J. Neurosci. 42, 4326–4341. 10.1523/JNEUROSCI.0987-21.2022 35477905 PMC9145239

[B53] Kingma B. R. Roijendijk L. M. Van Maanen L. Van Rijn H. Van Beurden M. H. (2021). Time perception and timed decision task performance during passive heat stress. Temperature 8, 53–63. 10.1080/23328940.2020.1776925 PMC7849768 33553505

[B54] Kitanishi T. Ujita S. Fallahnezhad M. Kitanishi N. Ikegaya Y. Tashiro A. (2015). Novelty-induced phase-locked firing to slow gamma oscillations in the hippocampus: requirement of synaptic plasticity. Neuron 86, 1265–1276. 10.1016/j.neuron.2015.05.012 26050043

[B55] Korotkova T. Ponomarenko A. Monaghan C. K. Poulter S. L. Cacucci F. Wills T.. (2018). Reconciling the different faces of hippocampal theta: the role of theta oscillations in cognitive, emotional and innate behaviors. Neurosci. Biobehav. Rev. 85, 65–80. 10.1016/j.neubiorev.2017.09.004 28887226

[B513] Kowalczyk T. Golebiewski H. Eckersdorf B. Konopacki J. (2001). Window effect of temperature on carbachol-induced theta-like activity recorded in hippocampal formation *in vitro*. Brain Res. 901, 184–194. 10.1016/s0006-8993(01)02355-1 11368966

[B56] Kraus B. J. Robinson II R. J. White J. A. Eichenbaum H. Hasselmo M. E. (2013). Hippocampal “time cells”: time versus path integration. Neuron 78, 1090–1101. 10.1016/j.neuron.2013.04.015 23707613 PMC3913731

[B57] Kristofferson A. B. (1966). A time constant involved in attention and neutral information processing (No. NASA-CR-427). 5295953

[B58] Kropff E. Carmichael J. E. Moser E. I. Moser M. B. (2021). Frequency of theta rhythm is controlled by acceleration, but not speed, in running rats. Neuron 109, 1029–1039.e8. 10.1016/j.neuron.2021.01.017 33567253 PMC7980093

[B59] Kunec S. Hasselmo M. E. Kopell N. (2005). Encoding and retrieval in the CA3 region of the hippocampus: a model of theta-phase separation. J. Neurophysiol. 94, 70–82. 10.1152/jn.00731.2004 15728768

[B60] Larkin M. C. Lykken C. Tye L. D. Wickelgren J. G. Frank L. M. (2014). Hippocampal output area CA1 broadcasts a generalized novelty signal during an object-place recognition task. Hippocampus 24, 773–783. 10.1002/hipo.22268 24596296 PMC4065199

[B508] Leutgeb S. Leutgeb J. K. Treves A. Moser M. B. Moser E. I. (2004). Distinct ensemble codes in hippocampal areas CA3 and CA1. Science 305, 1295–1298. 10.1126/science.1100265 15272123

[B62] Lever C. Burton S. Jeewajee A. Wills T. J. Cacucci F. Burgess N.. (2010). Environmental novelty elicits a later theta phase of firing in CA1 but not subiculum. Hippocampus 20, 229–234. 10.1002/hipo.20671 19623610 PMC3173854

[B61] Lever C. Burton S. ÓKeefe J. (2006). Rearing on hind legs, environmental novelty and the hippocampal formation. Rev. Neurosci. 17, 111–133. 10.1515/revneuro.2006.17.1-2.111 16703946

[B63] Lever C. Kaplan R. Burgess N. (2014). “The function of oscillations in the hippocampal formation,” in Space, Time and Memory in the Hippocampal Formation, eds Derdikman D. Knierim J. J. (Vienna: Springer), 303–350.

[B64] Lever C. Wills T. Cacucci F. Burgess N. O’Keefe J. (2002). Long-term plasticity in hippocampal place-cell representation of environmental geometry. Nature 416, 90–94. 10.1038/416090a 11882899

[B65] Lisman J. E. Grace A. A. (2005). The hippocampal-VTA loop: controlling the entry of information into long-term memory. Neuron 46, 703–713. 10.1016/j.neuron.2005.05.002 15924857

[B66] Lisman J. E. Idiart M. A. (1995). Storage of 7±2 short-term memories in oscillatory subcycles. Science 267, 1512–1515. 10.1126/science.7878473 7878473

[B67] Lopes-dos-Santos V. van de Ven G. M. Morley A. Trouche S. Campo-Urriza N. Dupret D. (2018). Parsing hippocampal theta oscillations by nested spectral components during spatial exploration and memory-guided behavior. Neuron 100, 940–952.e7. 10.1016/j.neuron.2018.09.031 30344040 PMC6277817

[B68] López-Madrona V. J. Pérez-Montoyo E. Álvarez-Salvado E. Moratal D. Herreras O. Pereda E.. (2020). Different theta frameworks coexist in the rat hippocampus and are coordinated during memory-guided and novelty tasks. eLife 9:e57313. 10.7554/eLife.57313 32687054 PMC7413668

[B69] Lubenov E. V. Siapas A. G. (2009). Hippocampal theta oscillations are travelling waves. Nature 459, 534–539. 10.1038/nature08010 19489117

[B70] MacDonald C. J. Lepage K. Q. Eden U. T. Eichenbaum H. (2011). Hippocampal “time cells” bridge the gap in memory for discontiguous events. Neuron 71, 737–749. 10.1016/j.neuron.2011.07.012 21867888 PMC3163062

[B71] Manns J. R. Zilli E. A. Ong K. C. Hasselmo M. E. Eichenbaum H. (2007). Hippocampal CA1 spiking during encoding and retrieval: relation to theta phase. Neurobiol. Learn. Mem. 87, 9–20. 10.1016/j.nlm.2006.05.007 16839788

[B72] Matell M. S. Meck W. H. (2004). Cortico-striatal circuits and interval timing: coincidence detection of oscillatory processes. Cogn. Brain Res. 21, 139–170. 10.1016/j.cogbrainres.2004.06.012 15464348

[B73] McNaughton N. Kocsis B. Hajos M. (2007). Elicited hippocampal theta rhythm: a screen for anxiolytic and procognitive drugs through changes in hippocampal function?. Behav. Pharmacol. 18, 329–346. 10.1097/FBP.0b013e3282ee82e3 17762505

[B518] McNaughton N. Ruan M. Woodnorth M. A. (2006). Restoring theta-like rhythmicity in rats restores initial learning in the Morris water maze. Hippocampus 16, 1102–1110. 10.1002/hipo.20235 17068783

[B74] Meck W. H. Church R. M. Olton D. S. (1984). Hippocampus, time and memory. Behav. Neurosci. 98, 3–22. 10.1037/0735-7044.98.1.3 6696797

[B75] Meck W. H. Church R. M. Wenk G. L. Olton D. S. (1987). Nucleus basalis magnocellularis and medial septal area lesions differentially impair temporal memory. J. Neurosci. 7, 3505–3511. 10.1523/JNEUROSCI.07-11-03505.1987 3681402 PMC6569024

[B500] Meck W. H. (2005). Neuropsychology of timing and time perception. Brain Cogn. 58, 1–8. 10.1016/j.bandc.2004.09.004 15878722

[B76] Miall C. (1989). The storage of time intervals using oscillating neurons. Neural Comput. 1, 359–371. 10.1162/neco.1989.1.3.359

[B77] Mizuseki K. Sirota A. Pastalkova E. Buzsáki G. (2009). Theta oscillations provide temporal windows for local circuit computation in the entorhinal-hippocampal loop. Neuron 64, 267–280. 10.1016/j.neuron.2009.08.037 19874793 PMC2771122

[B78] Monaghan C. K. Chapman IV G. W. Hasselmo M. E. (2017). Systemic administration of two different anxiolytic drugs decreases local field potential theta frequency in the medial entorhinal cortex without affecting grid cell firing fields. Neuroscience 364, 60–70. 10.1016/j.neuroscience.2017.08.056 28890051 PMC5786889

[B79] Moran R. J. Campo P. Maestu F. Reilly R. B. Dolan R. J. Strange B. A. (2010). Peak frequency in the theta and alpha bands correlates with human working memory capacity. Front. Hum. Neurosci. 4:200. 10.3389/fnhum.2010.00200 21206531 PMC3009479

[B519] Mouchati P. R. Kloc M. L. Holmes G. L. White S. L. Barry J. M. (2020). Optogenetic “low theta” pacing of the septohippocampal circuit is sufficient for spatial goal finding and is influenced by behavioral state and cognitive demand. Hippocampus 30, 1167–1193. 10.1002/hipo.23248 32710688 PMC7902092

[B80] Muller R. U. Kubie J. L. (1987). The effects of changes in the environment on the spatial firing of hippocampal complex-spike cells. J. Neurosci. 7, 1951–1968. 10.1523/JNEUROSCI.07-07-01951.1987 3612226 PMC6568940

[B81] Newman E. L. Gillet S. N. Climer J. R. Hasselmo M. E. (2013). Cholinergic blockade reduces theta-gamma phase amplitude coupling and speed modulation of theta frequency consistent with behavioral effects on encoding. J. Neurosci. 33, 19635–19646. 10.1523/JNEUROSCI.2586-13.2013 24336727 PMC3858632

[B82] Ning W. Bladon J. H. Hasselmo M. E. (2022). Complementary representations of time in the prefrontal cortex and hippocampus. Hippocampus 33, 577–596. 10.1002/hipo.23451 35822589 PMC9444055

[B83] Nitz D. McNaughton B. (2004). Differential modulation of CA1 and dentate gyrus interneurons during exploration of novel environments. J. Neurophysiol. 91, 863–872. 10.1152/jn.00614.2003 14523073

[B84] O’Hanlon J. F. McGrath J. J. McCauley M. E. (1974). Body temperature and temporal acuity. J. Exp. Psychol. 102, 788–794. 10.1037/h0036321 4847736

[B85] O’Keefe J. Nadel L. (1978). The Hippocampus as a Cognitive Map. Oxford: Oxford University Press.

[B86] O’Keefe J. Recce M. L. (1993). Phase relationship between hippocampal place units and the EEG theta rhythm. Hippocampus 3, 317–330. 10.1002/hipo.450030307 8353611

[B87] Olton D. S. Meck W. H. Church R. M. (1987). Separation of hippocampal and amygdaloid involvement in temporal memory dysfunctions. Brain Res. 404, 180–188. 10.1016/0006-8993(87)91369-2 3567565

[B88] Park J. Lee H. Kim T. Park G. Y. Lee E. M. Baek S.. (2014). Role of low-and high-frequency oscillations in the human hippocampus for encoding environmental novelty during a spatial navigation task. Hippocampus 24, 1341–1352. 10.1002/hipo.22315 24910318

[B89] Pastalkova E. Itskov V. Amarasingham A. Buzsaki G. (2008). Internally generated cell assembly sequences in the rat hippocampus. Science 321, 1322–1327. 10.1126/science.1159775 18772431 PMC2570043

[B90] Patel J. Fujisawa S. Berényi A. Royer S. Buzsáki G. (2012). Traveling theta waves along the entire septotemporal axis of the hippocampus. Neuron 75, 410–417. 10.1016/j.neuron.2012.07.015 22884325 PMC3427387

[B91] Penley S. C. Hinman J. R. Long L. L. Markus E. J. Escabí M. A. Chrobak J. J. (2013). Novel space alters theta and gamma synchrony across the longitudinal axis of the hippocampus. Front. Syst. Neurosci. 7:20. 10.3389/fnsys.2013.00020 23805081 PMC3691506

[B92] Pentkowski N. S. Blanchard D. C. Lever C. Litvin Y. Blanchard R. J. (2006). Effects of lesions to the dorsal and ventral hippocampus on defensive behaviors in rats. Eur. J. Neurosci. 23, 2185–2196. 10.1111/j.1460-9568.2006.04754.x 16630065

[B93] Pfeiffer B. E. (2022). Spatial learning drives rapid goal representation in hippocampal ripples without place field accumulation or goal-oriented theta sequences. J. Neurosci. 42, 3975–3988. 10.1523/JNEUROSCI.2479-21.2022 35396328 PMC9097771

[B94] Poulter S. Hartley T. Lever C. (2018). The neurobiology of mammalian navigation. Curr. Biol. 28, R1023–R1042. 10.1016/j.cub.2018.05.050 30205053

[B95] Poulter S. Lee S. A. Dachtler J. Wills T. J. Lever C. (2021). Vector trace cells in the subiculum of the hippocampal formation. Nat. Neurosci. 24, 266–275. 10.1038/s41593-020-00761-w 33349710 PMC7116739

[B517] Prut L. Belzung C. (2003). The open field as a paradigm to measure the effects of drugs on anxiety-like behaviors: a review. Eur. J. Pharmacol. 463, 3–33. 10.1016/s0014-2999(03)01272-x 12600700

[B96] Quirk C. R. Zutshi I. Srikanth S. Fu M. L. Devico Marciano N. Wright M. K.. (2021). Precisely timed theta oscillations are selectively required during the encoding phase of memory. Nat. Neurosci. 24, 1614–1627. 10.1038/s41593-021-00919-0 34608335 PMC8556344

[B520] Rayan A. Donoso J. R. Mendez-Couz M. Dolón L. Cheng S. Manahan Vaughan D. (2022). Learning shifts the preferred theta phase of gamma oscillations in CA1. Hippocampus 32, 695–704. 10.1002/hipo.23460 35920344

[B521] Ross T. W. Easton A. (2022). The hippocampal horizon: constructing and segmenting experience for episodic memory. Neurosci. Biobehav. Rev. 132, 181–196. 10.1016/j.neubiorev.2021.11.038 34826509

[B97] Sabariego M. Tabrizi N. S. Marshall G. J. McLagan A. N. Jawad S. Hales J. B. (2021). In the temporal organization of episodic memory, the hippocampus supports the experience of elapsed time. Hippocampus 31, 46–55. 10.1002/hipo.23261 32956520

[B98] Sakata S. (2006). Timing and hippocampal theta in animals. Rev. Neurosci. 17, 157–162. 10.1515/revneuro.2006.17.1-2.157 16703949

[B99] Salz D. M. Tiganj Z. Khasnabish S. Kohley A. Sheehan D. Howard M. W.. (2016). Time cells in hippocampal area CA3. J. Neurosci. 36, 7476–7484. 10.1523/JNEUROSCI.0087-16.2016 27413157 PMC4945667

[B100] Santos-Pata D. Zucca R. Amil A. F. Principe A. Pérez-Enríquez C. Rocamora R.. (2022). Theta oscillations in the human hippocampus normalize the information content of episodic memory. bioRxiv [Preprint]. 10.1101/2022.06.27.497705

[B101] Scheffer-Teixeira R. Belchior H. Caixeta F. V. Souza B. C. Ribeiro S. Tort A. B. (2012). Theta phase modulates multiple layer-specific oscillations in the CA1 region. Cereb. Cortex 22, 2404–2414. 10.1093/cercor/bhr319 22079925

[B102] Schomburg E. W. Fernández-Ruiz A. Mizuseki K. Berényi A. Anastassiou C. A. Koch C.. (2014). Theta phase segregation of input-specific gamma patterns in entorhinal-hippocampal networks. Neuron 84, 470–485. 10.1016/j.neuron.2014.08.051 25263753 PMC4253689

[B103] Shimbo A. Izawa E. I. Fujisawa S. (2021). Scalable representation of time in the hippocampus. Sci. Adv. 7:eabd7013. 10.1126/sciadv.abd7013 33536211 PMC7857679

[B104] Siegle J. H. Wilson M. A. (2014). Enhancement of encoding and retrieval functions through theta phase-specific manipulation of hippocampus. eLife 3:e03061. 10.7554/eLife.03061 25073927 PMC4384761

[B105] Siok C. J. Taylor C. P. Hajos M. (2009). Anxiolytic profile of pregabalin on elicited hippocampal theta oscillation. Neuropharmacology 56, 379–385. 10.1016/j.neuropharm.2008.09.013 18930748

[B106] Skaggs W. E. McNaughton B. L. Wilson M. A. Barnes C. A. (1996). Theta phase precession in hippocampal neuronal populations and the compression of temporal sequences. Hippocampus 6, 149–172. 10.1002/(SICI)1098-1063(1996)6:2<149::AID-HIPO6>3.0.CO;2-K 8797016

[B503] Stroud J. M. (1955). “The fine structure of psychological time,” in Information Theory in Psychology, ed Quastler H. (Glencoe), 111.

[B107] Tam S. K. Bonardi C. (2012). Dorsal hippocampal involvement in appetitive trace conditioning and interval timing. Behav. Neurosci. 126, 258–269. 10.1037/a0027164 22352787

[B108] Tamm M. Jakobson A. Havik M. Burk A. Timpmann S. Allik J.. (2014). The compression of perceived time in a hot environment depends on physiological and psychological factors. Q. J. Exp. Psychol. (Hove) 67, 197–208. 10.1080/17470218.2013.804849 23768002

[B109] Trimper J. B. Stefanescu R. A. Manns J. R. (2014). Recognition memory and theta-gamma interactions in the hippocampus. Hippocampus 24, 341–353. 10.1002/hipo.22228 24227610 PMC4004109

[B110] Umbach G. Kantak P. Jacobs J. Kahana M. Pfeiffer B. E. Sperling M.. (2020). Time cells in the human hippocampus and entorhinal cortex support episodic memory. Proc. Natl. Acad. Sci. U S A 117, 28463–28474. 10.1073/pnas.2013250117 33109718 PMC7668099

[B111] van Maanen L. Van der Mijn R. van Beurden M. H. Roijendijk L. M. Kingma B. R. Miletić S.. (2019). Core body temperature speeds up temporal processing and choice behavior under deadlines. Sci. Rep. 9:10053. 10.1038/s41598-019-46073-3 31296893 PMC6624282

[B501] Vidalaki V. N. Ho M.-Y. Bradshaw C. M. Szabadi E. (1999). Interval timing performance in temporal lobe epilepsy: differences between patients with left and right hemisphere foci. Neuropsychologia 37, 1061–1070. 10.1016/s0028-3932(98)00155-9 10468369

[B112] Villette V. Poindessous-Jazat F. Simon A. Léna C. Roullot E. Bellessort B.. (2010). Decreased rhythmic GABAergic septal activity and memory-associated θ oscillations after hippocampal amyloid-β pathology in the rat. J. Neurosci. 30, 10991–11003. 10.1523/JNEUROSCI.6284-09.2010 20720106 PMC6633464

[B113] Vosskuhl J. Huster R. J. Herrmann C. S. (2015). Increase in short-term memory capacity induced by down-regulating individual theta frequency via transcranial alternating current stimulation. Front. Hum. Neurosci. 9:257. 10.3389/fnhum.2015.00257 26005411 PMC4424841

[B504] Wang M. Foster D. J. Pfeiffer B. E. (2020). Alternating sequences of future and past behavior encoded within hippocampal theta oscillations. Science 370, 247–250. 10.1126/science.abb4151 33033222 PMC8594055

[B114] Wang Y. Romani S. Lustig B. Leonardo A. Pastalkova E. (2015). Theta sequences are essential for internally generated hippocampal firing fields. Nat. Neurosci. 18, 282–288. 10.1038/nn.3904 25531571

[B115] Wearden J. H. Penton-Voak I. S. (1995). Feeling the heat: body temperature and the rate of subjective time, revisited. Q. J. Exp. Psychol. B 48, 129–141. 10.1080/14640749508401443 7597195

[B116] Wells C. E. Amos D. P. Jeewajee A. Douchamps V. Rodgers J. O’Keefe J.. (2013). Novelty and anxiolytic drugs dissociate two components of hippocampal theta in behaving rats. J. Neurosci. 33, 8650–8667. 10.1523/JNEUROSCI.5040-12.2013 23678110 PMC3715395

[B512] Whishaw I. Q. Vanderwolf C. H. (1971). Hippocampal EEG and behavior: effects of variation in body temperature and relation of EEG to vibrissae movement, swimming and shivering. Physiol. Behav. 6, 391–397. 10.1016/0031-9384(71)90172-7 5148749

[B117] Wills T. J. Lever C. Cacucci F. Burgess N. O’Keefe J. (2005). Attractor dynamics in the hippocampal representation of the local environment. Science 308, 873–876. 10.1126/science.1108905 15879220 PMC2680068

[B118] Wolinski N. Cooper N. R. Sauseng P. Romei V. (2018). The speed of parietal theta frequency drives visuospatial working memory capacity. PLoS Biol. 16:e2005348. 10.1371/journal.pbio.2005348 29538384 PMC5868840

[B119] Yeung M. Dickson C. T. Treit D. (2013). Intrahippocampal infusion of the Ih blocker ZD7288 slows evoked theta rhythm and produces anxiolytic-like effects in the elevated plus maze. Hippocampus 23, 278–286. 10.1002/hipo.22086 23280856

[B120] Yeung M. Treit D. Dickson C. T. (2012). A critical test of the hippocampal theta model of anxiolytic drug action. Neuropharmacology 62, 155–160. 10.1016/j.neuropharm.2011.06.011 21723303

[B121] Yin B. Meck W. H. (2014). Comparison of interval timing behaviour in mice following dorsal or ventral hippocampal lesions with mice having δ-opioid receptor gene deletion. Philos. Trans. R. Soc. B Biol. Sci. 369:20120466. 10.1098/rstb.2012.0466 24446500 PMC3895991

[B122] Yin B. Troger A. B. (2011). Exploring the 4th dimension: hippocampus, time and memory revisited. Front. Integr. Neurosci. 5:36. 10.3389/fnint.2011.00036 21886612 PMC3154297

[B516] Young C. K. McNaughton N. (2020). Mixed effects of low-dose ethanol on cortical and hippocampal theta oscillations. Neuroscience 429, 213–224. 10.1016/j.neuroscience.2020.01.007 31954825

[B123] Zhang H. Jacobs J. (2015). Traveling theta waves in the human hippocampus. J. Neurosci. 35, 12477–12487. 10.1523/JNEUROSCI.5102-14.2015 26354915 PMC4563037

